# Proteotransciptomics of the Most Popular Host Sea Anemone *Entacmaea quadricolor* Reveals Not All Toxin Genes Expressed by Tentacles Are Recruited into Its Venom Arsenal

**DOI:** 10.3390/toxins16020085

**Published:** 2024-02-05

**Authors:** Cassie M. Hoepner, Zachary K. Stewart, Robert Qiao, Emily K. Fobert, Peter J. Prentis, Alex Colella, Tim Chataway, Karen Burke da Silva, Catherine A. Abbott

**Affiliations:** 1 College of Science and Engineering, Flinders University, Bedford Park, SA 5042, Australia; 2 Centre for Agriculture and Bioeconomy, Queensland University of Technology, Brisbane, QLD 4001, Australia; 3 School of BioSciences, University of Melbourne, Melbourne, VIC 3010, Australia; 4 Flinders Proteomics Facility, College of Medicine and Public Health, Flinders University, Bedford Park, SA 5042, Australia

**Keywords:** proteotranscriptomics, sea anemone, symbiosis, toxin, venomics

## Abstract

While the unique symbiotic relationship between anemonefishes and sea anemones is iconic, it is still not fully understood how anemonefishes can withstand and thrive within the venomous environment of their host sea anemone. In this study, we used a proteotranscriptomics approach to elucidate the proteinaceous toxin repertoire from the most common host sea anemone, *Entacmaea quadricolor*. Although 1251 different toxin or toxin-like RNA transcripts were expressed in *E. quadricolor* tentacles (0.05% of gene clusters, 1.8% of expression) and 5375 proteins were detected in milked venom, only 4% of proteins detected in venom were putative toxins (230), and they only represent on average 14% of the normalised protein expression in the milked venom samples. Thus, most proteins in milked venom do not appear to have a toxin function. This work raises the perils of defining a dominant venom phenotype based on transcriptomics data alone in sea anemones, as we found that the dominant venom phenotype differs between the transcriptome and proteome abundance data. *E. quadricolor* venom contains a mixture of toxin-like proteins of unknown and known function. A newly identified toxin protein family, Z3, rich in conserved cysteines of unknown function, was the most abundant at the RNA transcript and protein levels. The venom was also rich in toxins from the Protease S1, Kunitz-type and PLA2 toxin protein families and contains toxins from eight venom categories. Exploring the intricate venom toxin components in other host sea anemones will be crucial for improving our understanding of how anemonefish adapt to the venomous environment.

## 1. Introduction

All species from the Phylum Cnidaria (corals, sea anemones and jellyfish) are venomous, relying on toxins for defence, intra- and interspecific aggression and food acquisition [1]. Sea anemones, like other cnidarians, are the only venomous organisms that do not have a centralised venom gland (e.g., snakes, scorpions, octopuses [2]); instead, the venom is produced in tissues throughout their body via cnidocytes and ectodermal gland cells [1], with the venom containing a complex mixture of small molecules, peptides and proteins [3]. While the venom of sea anemones has begun to be characterised, the knowledge base of sea anemone toxins is still significantly behind what is known for other species, such as snakes and spiders, with very few studies utilising genomic, transcriptomics or proteomics methods [3,4,5,6]. Sea anemone genomic and transcriptomics studies suggest that venom contains proteins from numerous toxin families that are structurally and functionally diverse and are mostly under purifying selection [4,7,8]. There are approximately 1170 sea anemone species [9], yet only ten specific species of sea anemones from three unrelated anemone families (*Thalassianthiade*, *Actinidae*, *Stichodactylidae*) [10], form associations (as hosts) with one or more of the 28 species of anemonefishes (*Amphiprion*) [11,12,13]. Using a transcriptomics approach, Smith, et al. [14] determined that the venom phenotype of the sea anemone *Nematostella vectensis* (a non-host) may change quickly with expression of a single dominant toxin family enabling ecological specialization in this species. Expression of dominant toxins may consequently enable widespread ecological functions and thus may act convergently amongst animals with similar niches or behaviours. Dutertre et al. [15] combined venom duct transcriptomics and proteomics to discover that cone snails can rapidly produce and release two distinctive venom types depending on whether prey or predators are encountered. Similarly, using transcriptomics only, sea anemones have been shown to have distinctive toxin gene expression profiles in different tissue types [16,17] as they do not have a centralised venom gland. It is not yet known whether there are differences in toxin expression between host and non-host sea anemones which may have contributed to the evolution of symbiosis between host sea anemones and anemonefishes.

The host sea anemone provides a safe site for anemonefish reproduction and protection from predation [18], whereas the anemonefishes help to increase the growth, reproduction and defence of host sea anemones by providing nutrients from their faeces and increased oxygenation by swimming amongst the sea anemone’s tentacles and chasing off potential predators [19,20,21]. The venom of host sea anemones is understudied with only a few unique proteins/peptide sequences available in sequence databases compared to those available for both non-host sea anemones and other venomous species (7579 toxin sequences reported in Tox-Prot, of which 288 toxin sequences are from Actiniaria, as of December 2023 [22]). There are only 58 toxin sequences reported in Tox-Prot (as of December 2023 [22]), from seven of the ten host sea anemone species, indicating how little knowledge is available on the venom arsenal of host sea anemones. Nedosyko et al. [23] demonstrated variations in toxicity among the ten host sea anemones, with host sea anemones with a middle range toxicity forming more anemonefish associations than host sea anemones with a high or low toxicity. However, it is unclear how toxic host sea anemone venom is compared to non-host sea anemone venom or the influence of symbiosis on host sea anemone toxin and venom production.

The study of sea anemone venom has begun to use a combined transcriptomics and proteomics approach, also known as proteotranscriptomics [14,24,25,26,27,28]. Using proteotranscriptomics provides a more holistic overview of venom complexity, enabling the detection of novel proteins [24]. Three recent studies have utilised transcriptomics-only approaches focusing on host sea anemones that form associations with anemonefishes [29,30,31]. Delgado et al. [29] examined the toxin expression profiles of five host sea anemones and a closely related non-host, utilising existing transcriptomes in NCBI generated from different sea anemone tissues (outer and inner tentacles, column, exocodic and endocodic tentacles, etc.). Delgado et al. [29] inferred that haemostatic and haemorrhagic toxin gene expression is a dominant feature of host sea anemones. Barua et al. [31] and Kashimoto et al. [30] created new transcriptome datasets of host anemones from Okinawa, Japan to explore nematocyte expressed genes, phylogeny and co-expression in the evolution of sea anemones hosting anemonefish. Kashimoto et al. [30] noticed that nematocyte gene expression is generally uniform across host sea anemones, indicating that symbiosis is likely related to small gene or expression changes [32]. Barua et al. [31] observed that association with Symbiodiniaceae and anemonefishes significantly affect gene expression in host sea anemones, particularly in relation to nutrient exchange and metabolism. However, there has only been a single study on host sea anemone venom that has used a combined transcriptomics and proteomics approach [24]. Madio et al. [24] observed that there is a disparity between toxins expressed in the tentacle transcriptome compared to those recovered in the venom proteome, illustrating the importance of more direct proteomics investigations in this area in order to fully understand venom diversity and functionality.

The sea anemone species, *Entacmaea quadricolor*, forms the most associations with anemonefish species, 17 of 28 [33]. Nedosyko et al. [23] found that *E. quadricolor* was of mid-range toxicity compared to other host sea anemones and, together with its unique bulb-like tentacles, provides optimal conditions for anemonefishes. There are only three *E. quadricolor* toxin protein sequences reported in UniProtKB (as of December 2023 [22]), making it impossible to ascertain a complete picture of toxin characteristics and their evolutionary implications. To understand how anemonefishes can withstand their venomous host sea anemone environment, it is important to develop an in-depth profile of the venom to which the anemonefishes must develop resistance to. In this study, we expand upon the previous work of Barua et al. [31], Delgado et al. [29], Kashimoto et al. [30] and Madio et al. [24] to uncover the full toxin protein arsenal of *E. quadricolor* venom using a proteotranscriptomics approach.

## 2. Results

### 2.1. De Novo Tentacle Transcriptome Assembly

**Figure 1:**
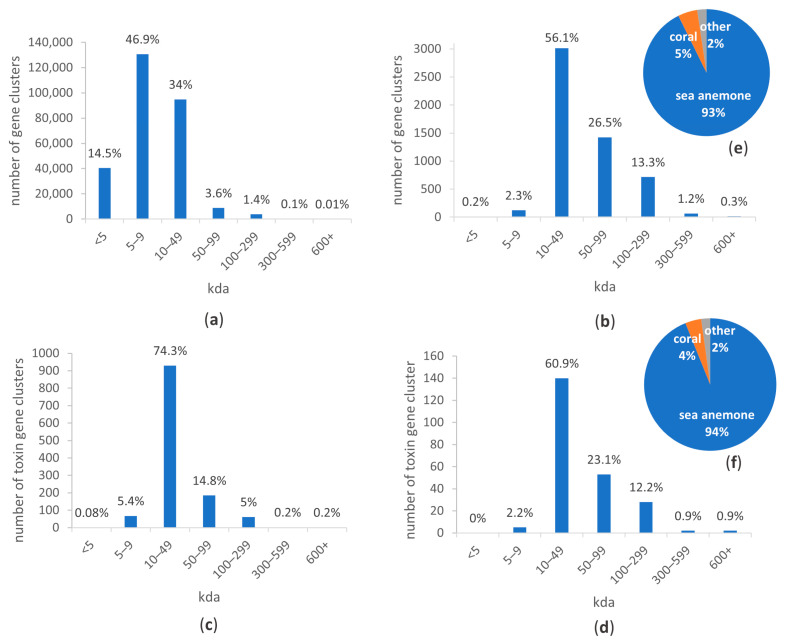
Size range of proteins identified in *Entacmaea quadricolor* based on (**a**) the amino acid translation of all tentacle gene clusters, (**b**) proteins identified in the venom, (**c**) the amino acid translation of toxin tentacle gene clusters, (**d**) toxin proteins identified in the venom. Pie chart of taxon from UniRef90 hit matches in (**e**) venom proteome and (**f**) toxins in venom proteome. Percentages are based on number of gene clusters in each sub-category (size range or species) divided by the total number of clusters in each category.

The de novo assembled tentacle transcriptome of *E. quadricolor* consisted of 650,353 ORFs after PsyTrans [34] symbiont contaminant removal. BUSCO scoring indicated that a high-quality assembly was achieved, with 98.3% of near-universal metazoan single-copy genes predicted (BUSCO short summary = Completeness: 98.3% [Single copy: 13.1%, Duplicates: 85.2%], Fragmented: 0.6%, Missing: 1.1%, *n*: 954). Clustering with Corset reduced the data to 279,274 gene clusters (Supplementary File S1), which substantially reduced the number of redundant transcripts (BUSCO short summary after clustering = Completeness: 94.0% [Single copy: 73.7%, Duplicates: 20.3%], Fragments: 1.5%, Missing: 4.5%, *n*: 95) (Table 1). Most gene clusters in the *E. quadricolor* tentacle transcriptome encoded proteins between 5 and 9 kda (46.9%) with only 5.1% of gene clusters encoding molecular weight proteins > 50 kDa (Figure 1a). Of the 279,275 gene clusters identified and translated, only 72,218 were annotated, matching to 18,469 unique proteins using the UniRef90 database (Table 1). Thus, over 74% of the *E. quadricolor* tentacle transcriptome represented novel transcripts that had no significant hits to proteins in UniRef90 (utilising a stringent E-value ≤ 1 × 10^−5^ for significance).

### 2.2. Venom Proteome

Using proteomics, a spectral library was created identifying proteins matching to 5375 gene clusters (1.9% of 279,274 gene clusters in the tentacle transcriptome) in milked *E. quadricolor* venom. Unlike the tentacle transcriptome, 97% of the proteins identified in the venom were annotated, matching to 3718 unique UniRef90 hits, with only 162 proteins having no known protein match (Table 1). Approximately 56% of proteins identified in venom were between 10 and 49 kda in size, and only 2.5% of proteins identified were <10 kda (compared to the 61.4% of gene clusters < 10 kda in the tentacle transcriptome) (Figure 1a,b). Ninety-three percent of proteins identified in the *E. quadricolor* venom proteome matched to proteins present in other sea anemones (Figure 1e). Specifically, in the venom proteome 77.4% of proteins (4161 proteins) matched genes identified in the non-host sea anemone *Actinia tenebrosa* genome [4].

### 2.3. Putative Toxins

Less than 0.05% of the *E. quadricolor* tentacle gene clusters were annotated as encoding putative toxins. By combining our automated toxin annotation pipeline with a manual search looking for protein families identified in Delgado et al. [29], a set of 1251 putative toxin gene clusters was created. This putative toxin set matched to 296 unique UniRef90 protein hits, with 22 gene clusters with no UniRef90 match (Table 1). Only 4% of proteins identified in the *E. quadricolor* venom proteome were assigned as putative toxins (proteins encoded by 230 gene clusters), 94% of which matched to toxins in other sea anemones (Figure 1f). Approximately 61% of the toxin proteins identified in venom were between 10 and 49 kda in size, while only 2.2% of toxin proteins identified were <10 kda (compared to the 5.44% of toxin gene clusters < 10 kda in the tentacle transcriptome) (Figure 1c,d, Supplementary Files S2 and S3). This putative toxin set matched to 124 unique UniRef90 protein hits, with all 22 gene clusters with no UniRef90 match from the transcriptome present in the venom. There were 180 *A. tenebrosa* protein orthologs identified as toxins in the *E. quadricolor* milked venom (78.3%).

### 2.4. Toxin Tentacle Gene Clusters Detected in Venom

The milked venom proteome only contained 18.4% of the gene clusters classified as toxins in the tentacle transcriptome. The allergen and innate immunity venom category had the highest number of gene clusters detected in venom, with 44.4% of tentacle gene clusters from this category being translated into proteins that were detected in the venom, with gene clusters from two out of three toxin family categories present (Table 2). However, while we identified all five toxin families from the haemostatic and haemorrhagic toxin category present in the tentacle transcriptome to be also present in the venom proteome, only 13.4% of gene clusters from this category are translated and transported into the milked venom arsenal. Mixed function enzymes had the lowest rate of gene transcripts appearing as proteins in the venom proteome, with only 4.2% (1 gene cluster), and neurotoxins had the lowest rate of toxin protein families transferred between the transcriptome and proteome, with only five out of the nine neurotoxin toxin families identified in the transcriptome present in the venom proteome.

### 2.5. Exploration of the Dominant E. quadricolor Venom Phenotype

**Figure 2:**
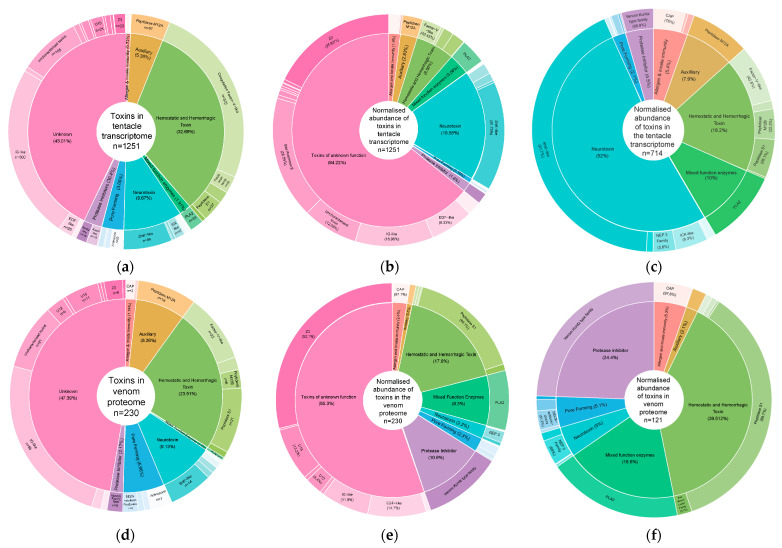
Pie donuts visualising venom function category (% = percentage of venom composition) (inner circle) and toxin protein family (*n* = number of gene clusters in each family) (outer circle) for (**a**) gene clusters from tentacle transcripts, (**b**) normalised abundance from tentacle transcripts, (**c**) normalised abundance from tentacle transcripts without toxins of unknown function category, (**d**) gene clusters from venom proteins, (**e**) normalised abundance from venom proteins, (**f**) normalised abundance from venom proteins without toxins of unknown function category.

In our *E. quadricolor* transcriptome, putative toxin genes were assigned to eight venom function categories (including a venom category of “unknown”) and 41 toxin protein families. A subset of unknown venom category gene clusters (104) were annotated as putative toxins (Tables S1 and S3). This unknown function venom category accounted for 42.8% of gene clusters assigned as toxins in our pipeline, followed by haemostatic and haemorrhagic toxins accounting for 32.7% of gene clusters (Figure 2a). Neurotoxins and toxins of unknown function were the two most diverse venom protein function categories as they included representatives from 9 and 16 different toxin protein families, respectively (Table S1). DESeq2-normalised counts [35] were used to normalise transcript expression data across our 24 tentacle RNA-seq samples and produce transcript abundance data for each gene cluster. These normalised counts were averaged across the 24 samples and then summed for all gene clusters within a toxin protein family to obtain gene expression levels at the family level. This approach also demonstrated that toxins of unknown function were the major toxin family expressed in the tentacles (64%) (Figure 2b). Neurotoxins accounted for 18% of toxin gene transcript expression, such that they were the second most abundant toxin category at the transcript level, and this was driven by the ShK-like protein family transcripts (Figure 2b). When toxins of unknown function are excluded from our analysis, the ShK-like neurotoxins represent 42% of the normalised toxin gene counts in the tentacles. However, when we consider total normalised gene counts, toxin gene counts only represent a small fraction, 1.8%, of the gene transcripts expressed in tentacles.

While proteins from all eight venom function categories were identified in the proteome, these were only from 30 toxin protein families, and 21 of these proteins were putative toxins which were not assigned to a toxin protein family (Tables S1 and S3). Toxins of unknown function accounted for 47.4% of gene clusters appearing in the venom proteome, followed by haemostatic and haemorrhagic toxins, which accounted for 23.9% of the gene clusters that were detected in the venom (Figure 2c). Like with the tentacle transcript gene clusters, toxins of unknown function are the most diverse venom function categories observed in the milked venom, containing 11 different types of toxin protein families. Normalised protein expression data obtained by our DIA proteomics workflow showed that toxins of unknown function were the major toxin protein component in the venom proteome (55.3%). With the haemostatic and haemorrhagic toxin category accounting for 17.6% of toxin protein expression, it was the second most abundant toxin component (Figure 2e). If we exclude toxins of unknown function from our analysis, the peptidase S1 toxin family represents approximately 35% of the toxin abundance in the venom.

When we count the number of gene clusters in each venom category, the dominant toxin protein families observed in the tentacle transcriptome and venom proteome data are quite similar. With coagulation factor V-like toxins from the haemostatic and haemorrhagic category and IG-like toxins from the unknown category dominating in the transcriptome toxin gene clusters, these then swap position in the toxin gene clusters observed in the venom proteins (Figure 2a,d, Table 3). In addition, when counting the number of gene clusters observed in each protein family, eight of the toxin protein families ranked in the top 10 in both the transcriptome and proteome data.

When we look at toxin gene expression profiles quantitatively utilising normalised gene counts and proteomics mass abundance data for each gene cluster, the venom expression phenotype looks very different (Table 4). The Z3 protein family from the unknown toxin function category is now the most abundant toxin family at both the RNA and protein level. In contrast, sea anemone 8 toxin, which was the second most abundant toxin at the RNA level, is not detected in the venom proteins at all. The second most abundant toxin at the protein level (Peptidase S1) does not make the top ten most abundant toxin families at the RNA transcript level (Table 4). Similarly, the ShK-like neurotoxin protein family had the third highest gene expression at the RNA level but only ranked 18th when assessed for protein expression. Only four of the top ten toxin protein families based on gene transcript levels in the tentacle transcriptome data also appeared in the top ten toxin protein families based on their protein expression levels. If we look quantitatively at toxin protein abundance across our seven venom samples, toxin proteins represented between ten and 18% (average 14%) of the total proteins present in each milked venom sample, whereas across the 24 tentacle RNA seq samples, toxin gene clusters represented between 1.1 and 2.4% (average 1.8%) of the normalised gene counts present in each tentacle sample.

#### 2.5.1. Toxins Assigned to the Unknown Venom Function Category

Fifty to sixty percent of the toxin protein families that appear in the top ten when protein and RNA transcript levels are considered are from the unknown venom function category (Figure 2, Table 4, Table S1). This function category also features heavily when we consider the number of gene clusters identified in the *E. quadricolor* trancriptome. Twelve new disulfide-rich peptides/protein families, named U1 to U12, were first identified as novel sea anemone toxin scaffolds in a proteomics study into the venom of *Stichadactyla haddoni*, another host sea anemone [24]. U12 and U15-like proteins were detected in the *E. quadricolor* venom with high protein expression, but the function of these toxin families is still unknown. Z3 is another potential toxin protein family with a unique cysteine scaffold which is expressed at the RNA level and detected in venom in *A. tenebrosa* and *Telmatactis stephensoni* [36]. Z3 proteins were the most highly expressed toxin protein family at both the RNA (across 20 gene clusters) and protein levels (across 6 gene clusters) in this *E. quadricolor* study.

IG-like toxins also feature in the top ten most highly expressed toxins at both the RNA and protein levels. Moreover, 300 of these IG-like gene clusters were identified in the *E. quadricolor* transcriptome. The 56 IG-like proteins that were detected in the venom proteome consisted of 26 different architectures of IG-like domains (Table S2). Thirty-one of these architectures contained repeats of the IG-like domain ranging from one to 13, and one contained 71 repeating IG-like domains, an Src homology 2 (SH2), a Dbl homology and a PH domain. Twenty-one architectures contained additional functional domains, such as protein kinase, SEA (sperm protein, Enterokinase and Agrin), PTX-2 (Pentraxin), fibrinogen C-terminal and fibronectin type III. It is currently unclear what function that the IG-like domain proteins are performing in the venom, but this gene family is conserved and found in the venom of several sea anemone species [36]. Multiple sequence alignment showed that 17 of the IG-like proteins found in *E. quadricolor* venom share homology with venom inhibitor proteins identified in marsupial opossums, *Didelphis marsupiali* (DM43 and DM64) and *D. virginiana* (Alpha 1B-glycoprotein) (Figure S1). The *E. quadricolor* IG-like proteins are also homologous to proteins identified in other sea anemones, including *N. vectensis,* and have been annotated as hemicentin-like due to their homology with a very large protein secreted by *Caenorhabditis elegans* (worm), which is involved in cell adhesion and the extracellular matrix and contains 48 tandem IG repeat motifs [28].

As mentioned above, there are 21 gene clusters that were present in the venom that were assigned to the unknown venom category and were labelled uncharacterised toxins in our pipeline (Table S3). Many of these uncharacterised proteins contained one or more PROSITE protein domains [37] often found in toxins (e.g., Trypsin, C-type lectin, Astacin, Fa5Bc-3, etc.), but they could not be assigned to a single venom category using the toxin family models in our automatic pipeline. In addition, fifteen of these proteins also contained between one and six ShK-like domains. Two of the unknown function gene clusters shared 54% amino acid identity with each other but had no detectable PROSITE domains. Instead, they shared approx. 40% amino acid identity and a six-cysteine scaffold with a known neurotoxin, U-actitoxin-Aer2a, that causes crab lethality from the sea anemone *Anemonia erytraea* [38].

#### 2.5.2. Haemostatic and Haemorrhagic Venom Category

This venom category contained the second highest number of gene clusters (~33%, Figure 2a), which were assigned mostly to three toxin protein families: Coagulation factor V-like family (315), Peptidase S1 (37) and the Ficolin lectin family (30). A smaller subset of gene clusters from these toxin families were identified in the venom proteins in addition to members of the Peptidase M12 B family. While Coagulation factor V-like gene transcripts were the most abundant at the RNA transcript level (Figure 2b), Peptidase S1 was more abundant at the protein level (Figure 2e). Coagulation factor V-like proteins have been identified in venom from Australian elapid snakes, including *Pseudonaja textilis*, *Oxyuranus microlepidotus* and *Oxyuranus scutellatus* [39]. The venom factor V protein from *P. textilis* has been shown to have a strong procoagulant nature which can facilitate disseminated coagulation in envenomated prey. Despite 315 gene clusters from this toxin family being identified in tentacles, only 22 Coagulation factor V-like gene clusters were detected in *E. quadricolor* venom. All Peptidase S1 toxins identified in the venom proteome contained a trypsin domain containing the three catalytic site residues, His, Asp and Ser, except for Cluster-31550.26173 (Figure S2). These Peptidase S1 toxins share homology with known thrombin-like snake venom serine proteases such as asperase in *Bothrops asper* (Terciopelo) and gyroxin in *Crotalus durissus terrificus* (South American rattlesnake) (Figure S2).

#### 2.5.3. Protease Inhibitor Venom Category

Five out of nineteen venom Kunitz-type family gene clusters discovered in the transcriptome were identified in *E. quadricolor* venom, and combined, they were the third most abundant toxins present at the protein level. Kunitz family members contain six conserved cysteine residues that form a prototype signal for a pancreatic trypsin inhibitor, as seen in the bovine pancreatic trypsin inhibitor, which inhibits not only trypsin but kallikrein, chymotrypsin and plasmin [40]. All of these proteins also share homology with U-actitoxin-Avd3n, a Kunitz peptide from the snakelock anemone, *Anemonia viridis*, that has been shown to directly activate neuronal G protein-coupled inward-rectifier potassium (GIRK1/2) channels, with minor effects on Kv1.6 channels [41] (Figure S3). The protease inhibitor proteins in *E. quadricolor* venom also shared homology with a Kunitz-type serine protease inhibitor from the black mamba, *Dendroaspis polylepis*, which has demonstrated trypsin inhibition activity [42].

#### 2.5.4. Mixed Function Enzymes Venom Category

Twenty-four tentacle gene clusters were assigned as PLA2 toxin family members belonging to the mixed function enzyme venom category in the transcriptome. While this toxin family was in the top ten list based on gene cluster RNA transcript abundance levels, only one of these gene clusters, which had the highest gene expression levels of the 24 gene clusters, was detected in venom, representing nearly 19% of total toxin protein abundance for proteins of known function present in venom (Figure 2f, Table 4). This PLA-2 protein shares 44% amino acid identity and 59% similarity with A2-actitoxin-Usc2a, identified in the mottled sea anemone *Urticina crassicornis*, which lacks haemolytic and neurotoxic activities [43] (Figure S4). Snake venom PLA2s are found in the venom of almost all venomous snake families, and they are often the major venom component and have a variety of pharmacological effects in snake bite victims (i.e., neurotoxicity, myotoxicity, anticoagulant effects, cytotoxicity, cardiotoxicity and edema [44]). The only *E. quadricolor* PLA2 detected in venom shares identity with numerous snake venom PLA2s, but these have been shown to have diverse pharmacological effects based on multiple functional site differences, and not all of their pharmacological effects have been elucidated [44]. However, the *E. quadricolor* PLA2 shares 38% amino acid identity and 47% amino acid similarity with a PLA2 protein found in the Australian taipan snake, *Oxyuranus scutellatus’* venom been shown to have neurotoxicity and myotoxicity [45,46]. The PLA2 in *E. quadricolor* venom is probably catalytically active as it contains the His48/Asp99 dyad at the active site [47], but it lacks the C-terminal extension region that has been shown to be responsible for myotoxic effects (Figure S4).

#### 2.5.5. Neurotoxin Venom Category

Neurotoxins accounted for 52% of the well-characterised toxin tentacle transcripts expressed in the tentacle, and this category was dominated by the expression of ShK-like gene cluster transcripts. In the tentacle transcriptome, there were 85 gene clusters assigned as ShK-like neurotoxins with only 14 of these gene clusters being detected in the milked venom proteins. Eleven of these ShK-like toxins only contained a single ShK-like domain despite there being sequences with multiple ShK-like domains present in the tentacle transcriptome. Three other ShK-like proteins in the venom contained two ShK-like domains. All proteins assigned as ShK-like toxins identified in the venom proteome shared homology with *Stichodactyla helianthus* Kappa-stichotoxin-She3a with a conserved cysteine scaffold. This peptide is a known neurotoxin with effects on potassium channels (Figure S5). However, while ShK-like toxins were the third most abundant toxic component of the transcriptome based on transcript levels, they did not feature in the top ten families based on protein expression levels. Instead, the neurotoxin NEP 3 toxin protein family ranked at number ten, representing 3.45% of the well-characterised toxin proteins present in venom.

#### 2.5.6. Pore-Forming Venom Category

The first toxin identified in *E. quadricolor* in 1994 was a 20 kDa protein which caused haemolysis of human red blood cells [48]. The pore-forming category of toxins, in particular the actinoporin protein family, made the top ten ranking of gene clusters in both the transcriptome and the proteome (Table 3, Figure 2a,d); however, this category did not feature in the top 10 list when RNA and protein abundance was considered. In our pipeline, seven clusters in the venom proteome were labelled as actinoporins and two were labelled as DELTA-actitoxin-Ucs1a (Cluster-31550.115050 and Cluster-31550.115051). All these actinoporins aligned with actinoporins that have been identified and deposited into NCBI from host and non-host sea anemones (Figure S6).

## 3. Discussion

Using proteotranscriptomics, this study provides the first holistic overview of gene expression and venom proteins from the most utilised host sea anemone, *E. quadricolor*. Previously, most studies exploring the venom composition of sea anemones and other venomous species have only utilised transcriptomics approaches to profile probable toxins in venom. Here, we have provided further evidence to support the assertion that a combined proteomics and transcriptomics approach is essential to accurately profile venom composition [24,27,49,50], particularly in the context of symbiosis, as anemonefish interact with host sea anemone venom proteins rather than RNA transcripts.

### 3.1. Putative Toxins

We discovered a high number of toxin genes (1251 gene clusters from 41 toxin families) using our toxin pipeline in comparison to what has been published for other host sea anemone transcriptome studies, e.g., *Sticodactyla haddoni* (508 toxin transcripts from 23 families and 27 toxin proteins [24]), *Cryptodendrum adhaesivum* (118 toxin transcripts from 14 families), *Macrodactyla doreensis* (72 toxin transcripts from 13 families [16]). Delgado et al. [29] is the only other study to assess the complete toxin profile of *E. quadricolor*, finding 328 toxin transcripts from 37 families using six datasets from the NCBI database. We identified more toxin genes in our study due to both our experimental design and the toxin annotation pipeline utilised. Firstly, the increase in toxin and non-toxin gene clusters assembled was likely due to the extensive sequence coverage achieved in this study (NovaSeq S4 flow cell, paired end 2 × 150 bp to achieve an average coverage of 30 million reads per sample). The addition of biological replication (*n* = 6) both with and without anemonefish presence and the inclusion of two tentacle sampling timepoints (0 and 72 h post venom milking [24]) to allow the toxin arsenal to be replenished after milking would have also increased our sequencing depth and thus our ability to detect toxin gene clusters. 

Secondly, our pipeline was also quite different to that used by other researchers. We used a lower ORF cut-off ≥30 amino acids to include gene clusters in our de novo transcriptome and for consideration as toxins. Previous studies used a higher open reading frame (ORF) cut-off, e.g., ≥50–70 amino acids [16,29,30,31,51], which may limit the discovery of smaller-molecular-weight toxins and explain the larger toxin dataset we acquired in this study. A subset of proteins less than 7 kda (7 kda is equivalent to an approximately 70 aa amino acid cut off) would not have been recovered by other studies using the higher amino acid ORF cut-offs. We found that 41.9% of our gene clusters identified encoded proteins <7 kda, which accounted for 117,222 proteins in the tentacle transcriptome and 478 proteins in the venom proteome (including nine toxins). In addition, many pipelines exclude gene clusters that encode proteins without a signal peptide from their toxin annotation process. However, if the ORF prediction of a gene cluster is a few nucleotides off from the “true” start or if the N-terminal methionine required for the start of protein translation is missing from the gene cluster transcript, the signal peptide may not be predicted accurately. For this reason, we did not exclude gene clusters without a signal peptide, and this variation alone more than doubled the toxin gene clusters included in our analysis.

Another difference in our pipeline was the inclusion of 486 unknown toxin family clusters which were annotated to either uncharacterised toxins, IG-like, U or Z protein families in our pipeline. To our knowledge, these protein families have not been included or annotated as toxins in other sea anemone studies, including Delgado et al. [29]. The IG-like proteins are a very large family adding 300 gene clusters to our tentacle toxin transcript arsenal. A proteotranscriptomics study by Madio et al. [24] discovered 12 new protein families based on cysteine scaffolds and amino acid sequence similarity in the venom of the host anemone *S. haddoni*. We were able to identify 5 of the 12 novel protein families found by Madio et al. [24] in *S. haddoni* venom within the *E. quadricolor* tentacle transcriptome and identified three more (U2, U8, U9, U11, U12, U13, U15 and U16). The Z toxin family also has a unique cysteine scaffold and has been observed in other sea anemone RNA transcripts and in venom, so it was included in our analysis. Ultimately, one of the true measures of whether a gene cluster is truly a toxin or not is whether it is found in venom.

### 3.2. Venom Proteome

Proteotranscriptomics revealed 5375 proteins in the venom of the host sea anemone *E. quadricolor*. Thus, this study identified a much larger number of proteins compared to previous sea anemone venom proteome studies, e.g., *S. haddoni* (135 proteins [24]), *Anthopleura dowii* (156 proteins [25]) and *Bunodactis verrucosa* (413 proteins [52]). This increase in proteins detected is probably due to the improvement in MS technology in recent years and the ability now to detect proteins at low concentrations in samples. Only two percent of the gene clusters produced in the tentacle transcriptome were detected in venom. Thus, 98% of the tentacle gene clusters are involved in other essential sea anemone functions required for growth and survival. Only 4% of proteins detected in venom were putative toxins (230), and they only represent, on average, 14% of the normalised protein expression in the milked venom samples. Thus, most proteins in milked venom do not appear to have a toxin function. As mentioned above, in our pipeline we did not exclude proteins that did not have a signal peptide from being considered as toxins or venom proteins. In fact, only 22.8% of proteins identified in the venom proteome had a signal peptide detected in their translated coding sequence. Thus, excluding proteins based on the assumption that not having a signal peptide means the protein would not be secreted into milked venom may not be the best approach for identifying toxins in sea anemones from RNA-seq and proteomics data. The complex processes required for cnidarians to produce nematocysts and to package and deliver toxins is still not fully understood. Further, an aqueous environment may pose challenges not encountered by other land-based venomous species, and perhaps these additional proteins may be required for dispersing toxins, reducing dilution or maintaining the viscosity of venom and mucus [48]. Overall, we have revealed that the milked venom profile of sea anemones is more complex than previously thought and that not all proteins in the venom are toxins; in fact, toxins only make up a very small proportion of the venom arsenal.

Moreover, some of the uncharacterised toxins from the IG-like (56 proteins), U (61 proteins) and Z (7 proteins) protein families identified in our transcriptomics pipeline were detected in the venom proteome, which is further evidence that these proteins may function as toxins, which is discussed below. Only three U proteins (U2, U8 and U11) were not present in *E. quadricolor* venom with the U12 and U15 toxin families dominating in terms of protein expression levels. Interestingly, Madio, Undheim and King [24] detected U2, U3 and U12 proteins only in their venom proteome and did not find gene transcripts for these proteins in their *S. haddoni* tentacle transcriptome, suggesting that these genes are transcribed in a different tissue yet still arrive in *S. haddoni* venom. Due to our use of the spectral library to ID the proteins in the proteomics pipeline, we were unable to identify any toxins or other proteins in the venom proteome that were not found in the tentacle transcriptome as the protein amino acid sequence library used to ID the proteome was generated from our transcriptome library.

### 3.3. Where Are the Toxin Genes That Are Expressed Going?

Sea anemones are far less anatomically complex than other venomous species that have evolved modified salivary glands to control the production and dispersion of venom [2]. While it is known that sea anemone toxins are packaged into nematocysts that can be fired at prey, it has also been found in *N. vectensis* that sea anemones have ectodermal glands that secrete toxins [53]. It has also been shown that different toxin genes are expressed by different sea anemone tissue types [16,17]. Thus, it is clear from the literature that not all toxins expressed by sea anemones are destined to be packaged in nematocysts for firing. Only 18% of toxin gene clusters that were identified in the tentacle transcriptome were found in the venom proteome, with several toxin protein families not appearing in the milked venom proteome or having a significant reduction in the number of gene clusters, but all venom toxin function categories were present in both datasets. Gene expression is energetically costly; thus, if the toxin genes are being expressed, they must play an essential ecological role for the sea anemone [54]. Sea anemone tentacles are used to capture prey and defend against predators and are covered with a mucus; it is possible that some of these toxin transcripts are translated into proteins that are trafficked into the mucus. It is important in future work that the proteome of the mucus be determined. This is particularly important in relation to host sea anemones, as anemonefishes interact directly with the mucus secretions of their host by performing a range of acclimation behaviours, including touching host sea anemone tentacles with their tail, biting the tentacle tips, and continuous fanning of tentacles with their pectoral fins [55], to acclimate and then enter their host sea anemone and therefore directly interact with any toxins that may be secreted into the mucus. Accurately profiling what toxins are trafficked into nematocytes and what toxins are secreted into the mucus may provide further insight into the level of toxin resistance required by anemonefishes to withstand this venomous environment.

### 3.4. Dominant Venom Hypothesis

The dominant venom hypothesis proposed by Smith et al. [14] suggests that individual species of sea anemones are defined by a venom phenotype with one venom function or toxin family dominating at the gene transcript level. They claim that “For most sea anemones (17 out of 29), a single toxin family contributed to the majority of the venom expression phenotype and accounted for >50% of the total toxin expression”. The data we present in this paper from the *E. quadricolor* host sea anemone does not support this hypothesis at all.

Smith et al. [14] only presented data based on eight toxin protein families from two venom function categories: actinoporins, which are pore-forming proteins, and the neurotoxins NEP3, NEP6 and NaTx and KTx1, KTx2, KTx3 and KTx5. The *E. quadricolor* gene expression data presented by Smith et al. [14] indicated that actinoporins dominate, representing 73% of the toxin expression present and that NEP6 is not expressed at all. In our *E. quadricolor* gene expression data, we also find no expression of NEP6, and we find two or more gene clusters for the other seven toxin families examined by Smith et al. [14]: actinoporins (32), NEP3 (5), NaTx (2), KTx1 (85), KTx2 (19), KTx3 (2) and KTx5 (17). However, our data, considering these eight genes only, indicate that KTx1, the ShK-like toxin gene family, represents 77% of the gene transcript expression, and this protein family contains the highest number of gene clusters. As this single toxin family contributed to the majority of the venom expression phenotype and accounted for >50% of the total toxin expression transcript expression using the Smith et al. [14] approach, neurotoxins would be considered the *E. quadricolor* dominant venom phenotype. This result is in contrast to the Smith et al. data, which presented the actinoporin toxin family as the dominant phenotype for *E. quadricolor*. These conflicting results demonstrate that caution needs to be taken when applying this hypothesis across different data sets collected from different tissue types and geographic locations and using different toxin prediction/annotation pipelines. Moreover, it is clear from our overall data (Figure 2) that the dominant venom phenotype will differ depending on whether the number of gene clusters or the gene transcript normalised counts or protein levels derived from MS data are used as the factor under consideration. In addition, it also depends on what toxin families are included in the analysis. To date, much of the emphasis has been to focus on toxin families that have been structurally and functionally validated or their expression localised to epithelial gland cells and nematocysts [56] or have been functionally assayed in extracts from sea anemones; however, this analysis has only been performed for only a few species, with analysis into host sea anemone species lacking.

Revisiting our data, if we include toxins of unknown function in our analysis, this toxin category is dominant at the gene cluster level (Figure 2a) and when assessing RNA (Figure 2b) and protein levels (Figure 2e). Five of the top ten toxin protein families based on protein abundance in milked venom were from the toxins of the unknown function venom category, indicating that our current understanding of sea anemone venom composition and functionality is still very limited, and more studies are required to determine the function of these newly identified ‘toxins’. One representative from Madio et al.’s [24] novel U toxin protein family (U12), a new U family member (U15) and a novel Z toxin protein family found using our toxin pipeline (Z3) feature in the top ten abundant toxin families in the *E. quadricolor* venom proteome, with Z3 the most abundant toxin protein family both at the RNA and protein levels. These toxin families have been modelled due their unique cysteine scaffolds. These scaffolds can lead to disulfide bonds and contribute to protein/peptide folding and stability and are typically present in extracellular proteins, such as toxins [1]. While the ecological role that these proteins may play for sea anemones is unknown, they do appear to be conserved and unique to the species, and their presence in venom suggests they may be toxins, but their target of action and their role in prey and defence will require further research.

Immunoglobulin-like (IG-like) proteins are another highly abundant toxin identified where the function is unknown. IG-like proteins are included in our toxin pipeline under the assumption that genes that are highly expressed during venom regeneration (e.g., our 72 h post milking sample) and are highly conserved across sea anemone species may be putative toxins [24]. The IG-like domain is one of the most widespread domains in animals and appears to be involved in binding functions. They are found in proteins involved in the immune system; however, the C2-type domain, which is observed in the *E. quadricolor* venom proteins, is found in proteins like neural cell adhesion molecule 2 and several growth factor receptors [57]. While it is unclear without biological assays what the function of IG-like proteins is in the venom, IG-like proteins are the largest group of natural venom inhibitor proteins. IG-like gene superfamily proteins, such as oprin, AHF-1 and DMP43, are known to neutralise snake venom metalloendopeptidases (SVMPs) and phospholipases and are found in the plasma, serum or muscle of mammals, such as mongooses or opossum, that are resistant to some snakebites [58,59]. Natural venom inhibitors also allow venomous species, such as snakes, to be resistant to their own venom [60]. Conservation and expansion of IG-like proteins must have occurred in the *E. quadricolor* genome to enable the birth of the 300 IG-like gene clusters observed in the tenacle transcriptome and the 56 IG-like proteins appearing in its venom, suggesting that these proteins are important to sea anemone ecology and speciation. Perhaps this protein family may enable self-recognition, which prevents the firing of nematocytes when their tentacles touch [61]. Molecular mimicry of host sea anemone mucus is proposed as a mechanism by which anemonefishes form resistance to their sea anemone host toxins [33,61]; the transfer of IG-like proteins, present in tentacle mucus onto anemonefish’s skin could also facilitate this process and warrants further investigation. Thus, it is possible that these IG-like proteins may not be toxins but are important to the ecological function of venom or the successful expansion of sea anemones as a species.

If we exclude this large unknown venom category, neurotoxins are the dominant venom phenotype (representing 52% of gene expression) for *E. quadricolor* based on normalised gene expression counts. The dominant toxin protein family then becomes ShK-like toxins, which are known to target the KTx1 channel. ShK from the sea anemone *S. helianthus* has been used as a scaffold to develop a highly selective Kv1.3 blocking peptide ShK-186, which is undergoing clinical trials for treatment of autoimmune disease [62]. However, if we look at the level of protein abundance, the haemostatic and haemorrhagic venom category is the dominant venom family (representing 39.5% of toxin proteins in the venom based on MS data), and this is driven by expression of the Peptidase S1 toxin protein family, which represents 35% of toxin function protein expression if we exclude the unknown category. The trypsin-like serine proteases, the Peptidase S1 family, is present in snake venom and is known to act on components of the haemostatic system of their prey. Whether *E. quadricolor* uses this venom component to capture prey or defend themselves is unknown and warrants further investigation.

Recently, Delgado et al. [29] using the publicly available datasets in NCBI assembled transcriptomes for five host and one non-host sea anemones to provide data that haemostatic and haemorrhagic toxins were a dominant part of the venom phenotype of host sea anemones; however, this analysis was performed based on the number of gene clusters in each venom category. Utilising a similar approach on our data (Figure 2a), if we exclude the unknown toxins identified in our toxin pipeline, coagulation factor V-like toxins are a dominant toxin family component in the transcriptome (315 gene clusters) and proteome (22 gene clusters) from the gene cluster perspective, as was seen in Delgado et al. [29]. While the coagulation factor V-like gene transcripts are highly expressed at the RNA transcript level, Peptidase S1 is the second most abundant toxin component of the venom proteome present after the Z3 proteins. Despite genes of this protein family being well documented in sea anemones [24,29,63], haemostatic and haemorrhagic toxins have not been functionally assayed in the venom of sea anemones, and thus the potency and function of these proteins in the venom remains unclear. As discussed above, these trypsin-like toxins can cleave peptide bonds and are responsible for coordinating blood coagulation. In the non-host sea anemone *N*. *vectensis*, trypsin domains have been found to have many putative functions, including digestion, wound healing, and blood coagulation, with 17 lineages of trypsin being identified in the common cnidarian ancestor [1]. However, in comparison to snake venoms, there is a distinct lack of different serine protease families in the venom of sea anemones generally as these venomous linages evolved separately. The peptidase S1 serine protease family proteins in *E. quadricolor* venom share homology with a thrombin-like serine protease from the Central American pit viper *B. asper*. This protease displayed fibrinogen-clotting activity in vitro and defibrinating activity in vivo [64]. In addition, the *E. quadricolor* proteins also share homology with a gyroxin B1.3 from the South American rattlesnake. This gyroxin was able to convert fibrinogen to fibrin and increase the permeability of the blood barrier in mice [65] and showed moderate inhibitory activity on the human voltage-gated potassium channel (KV10.1) [66]. All but one of the Peptidase S1 proteases in *E. quadricolor* venom contain the trypsin catalytic triad (His-Asp-Ser), and thus their protease activity could be involved in the activation of other proteases in the venom, the activation of toxin precursors, or in the digestion of prey. But it is also possible that these proteases could be directly involved in the capture of prey. Next to no studies have examined the effects of the sea anemone on components of the coagulation cascade to see whether they are able to affect the haemostatic system of prey. Oliveira et al. [67] demonstrated sea anemone extracts from *Millepora alcicornis*, *S. helianthus*, *Plexaura homomalla*, *Bartholomea annulata* and *Condylactis gigantea* were able to inhibit haemorrhagic activity induced by *Bothrops moojeni* venom. Only the *C. gigantea* (body wall) extracts inhibited thrombin-induced coagulation [67]. Given the abundance of the haemostatic and haemorrhagic toxins detected in *E. quadricolor* venom and the abundance of these genes in other sea anemone transcriptomes, it would be useful to investigate haemostatic and haemorrhagic activities of sea anemone venom in more detail.

The Kunitz family proteins, which are in the protease inhibitor venom category, were the third most abundant toxins present at the protein level in *E. quadricolor* venom, and then one mixed function category protein PLA2 was fourth, representing 10.9% and 8.3% of protein abundance, respectively, if the unknown function category is considered, or 24.4% or 18.6% if it is not. Kunitz proteins found in other venoms have been demonstrated to inhibit trypsin [68] and also to act as a neuronal G protein-coupled inward-rectifiers of potassium (GIRK1/2) channels, with minor effects on Kv1.6 channels [41]. The Kunitz family proteins detected in *E. quadricolor* venom could affect prey directly such that they could inhibit serine proteases in the haemostatic system or block potassium channels to be potent neurotoxins. These Kunitz proteins could also directly inhibit the abundant Peptidase S1 proteins expressed endogenously in the *E. quadricolor* venom, but the ecological purpose of this is unclear. Snake venom PLA2s are the most studied mixed function enzymes, and in addition to their possible role in the digestion of prey due to their ability to degrade membrane phospholipids, they show a wide variety of pharmacological effects, including neurotoxicity, cardiotoxicity, and involvement in haemolysis and haemorrhage [69].

Due to the complexity of the venom composition of host sea anemone venom, it is unlikely that the anemonefishes develop resistance to each toxin component in the venom. If we are to consider the toxins of known function only, venom is rich in Protease S1 and Kunitz proteins and a single PLA2. Although these three belong to three different venom function categories, all three protein families have the ability to affect the coagulation system of prey and thus could potentially all be involved in haemolysis and haemorrhage [69]. Thus, developing resistance or disrupting this toxin pathway could be a key mechanism that anemonefishes could utilise to become resistant to the majority of their host sea anemone’s venom profile. This is a concept that is yet to be considered in the current literature with previous hypotheses narrowed down to: (1) anemonefish mucus molecularly mimics the composition of the host sea anemone’s mucus to disguise themselves amongst their tentacles or (2) anemonefish mucus lacks the trigger for the firing of the host sea anemone’s nematocytes [33]. The expansion of IG-like proteins in the tentacles and venom of *E. quadricolor* gives support to hypothesis (1), as IG-like proteins are suspected to aid in self-recognition in sea anemones, and if anemonefishes were able to incorporate these proteins into their own mucus layer, they could become disguised and minimise their exposure to host sea anemone nematocytes.

In summary, it is evident from our proteomics abundance data that *E. quadricolor* sea anemone venom is more of a mixture of venom categories and thus toxin functions, and no singular venom phenotype appears to be dominating. A more important concept to consider when investigating how toxins present in venom contribute to the ability of the sea anemone to participate in essential ecological roles related to predation and defense and symbiosis with host organisms is that toxins will have different potencies, such that protein abundance may also not be the best way to determine the effectiveness of venom or its combined toxin phenotype. It would be assumed during molecular evolution that following gene duplication and subsequent changes to the amino acid sequence of toxins, amino acid changes that improve the potency and/or the ability of a toxin to target more generally or any specific prey species will be favored. In addition, small amino acid changes to a protein that improve binding between active sites and substrates in prey, or that improve binding to prey membranes, will enable proteins to work at lower concentrations, and a toxin, despite being present at low abundance, may be highly potent. For example, exploration of the structural/activity relationship in the 35 aa ShK peptide K+-channel toxin from *S. helianthus*, has led to the development of a peptide which inhibits Kv1.3 at 34 pM and is 158-fold selective for Kv1.1 [62,70]. Thus, ShK-like toxin proteins, or any other toxin family protein that is at low protein abundance, may be very potent and be able to kill or scare away prey/predators at extremely low protein concentrations.

## 4. Conclusions

Our proteotranscriptomics analysis of the host sea anemone *E. quadricolor* demonstrates that this species does not have a dominant venom expression phenotype as no single toxin family contributed to the majority of the venom expression phenotype or accounted for >50% of the total toxin transcript expression. To evaluate the dominant venom phenotype theory more fully, there is a need for a more standardised approach to be applied across future sea anemone venom research, in particular within the methodology used to predict toxins. Furthermore, given our work demonstrates a different phenotype is predicted when protein abundance is assessed, it highlights the importance of proteomics in future sea anemone venom research, and that caution needs to be applied when using RNA transcript expression data alone as an accurate predictor of major toxin function in sea anemone venom [24], a methodology which is often used in snake and arthropod venom research. The ability to dissect out the venom gland in snakes probably results in the transcriptomics toxin profile matching their venom toxin profile better, unlike in sea anemones, which lack a centralised venom gland [1] and differ in toxin transcript composition depending on the tissue type sampled [16,17,51].

Understanding the different venom toxin components in host sea anemones such as *E. quadricolor* and how they function and contribute to the ecological success of host species is important to better understand the ability of anemonefishes to adapt to this venomous environment. Future studies should also investigate the venom profile of host sea anemone mucus as the mucus covering the host sea anemone’s tentacles is the primary surface that anemonefishes interact with.

## 5. Materials and Methods

### 5.1. Study Species and Experimental Set-Up

Six sea anemones (*E. quadricolor*) (~5–7 cm diameter) were obtained from an aquarium store in Adelaide, South Australia (harvested from Western Australia) and transported to the Animal House facility at Flinders University and held in individual 30 L tanks for a 2-week acclimation period (26.5 °C ± 0.7, salinity 37.5 ± 1.5, pH 7.91 ± 0.2). *E. quadricolor* were fed a small piece of prawn every three to four days throughout the experimental period except in the 48 h leading up to each venom sampling event. Each tank had Fluval Aquatic Marine Nano 3.0 lights (2500 lux on a 12:12 L:D light cycle). Six pairs of anemonefish (*n* = 12) (*Amphiprion percula*) were housed in 30 L tanks containing a terracotta pot that acts as a sea anemone surrogate when a sea anemone was absent. Recirculating tanks (30 L) holding pairs of the *A. percula* were attached to a sump system separate from the sea anemones (27 °C ± 0.6, salinity 36.5 ± 1.5, pH 8.01 ± 0.2). The fish were fed twice daily with commercial pellets (Hikari Marine S) and mysid shrimp.

### 5.2. Sea Anemone Venom and Tentacle Collection

The *E. quadricolor* sea anemones were starved for 48 h prior to tenacle and venom sampling. Three tentacle samples were cut from each *E. quadricolor* individual (*n* = 6) during the non-hosting period (when not in association with an anemonefish) by stretching out the tentacle with sterile tweezers and slicing the tentacle at the base with a disposable scalpel. The tentacles were immediately placed in 400 µL of RNAlater (Sigma Aldrich, St. Louis, MI, USA) and stored at −80 °C. Each sea anemone was subsequently milked for venom, as described by Sencic and Macek [71] and Hoepner et al. [72], and the venom was freeze-dried and then stored at −80 °C. An additional three tentacles were collected 72 h after venom milking [24]. A pair of *A. percula* anemonefish were added to each tank after tentacle removal for a three-week acclimation period. Tentacle and venom sampling was repeated after three weeks of hosting fish.

### 5.3. Transcriptomics

#### 5.3.1. RNA Isolation and Library Preparation

RNA was extracted from the *E. quadricolor* tentacles (non-hosting 0 h, *n* = 6; non-hosting 72 h, *n* = 6; hosting 0 h, *n* = 6; hosting 72 h, *n* = 6) using an RNeasy mini kit (Qiagen Venlo, Limburg, The Netherlands) as per manufacturer’s instructions. In brief, tissue samples (*n* = 24) from each sampling point were disrupted using a mortar and pestle and ground to a fine powder under liquid nitrogen and added to the lysate buffer RLT and homogenised. The samples were then transferred to the mini spin column and the column was washed three times with wash buffer by centrifugation to remove contaminants. The RNA was eluted from the column using RNAse-free water before being stored at −80 °C. RNA was quality-controlled and quantified via LabChip (PerkinElmer, Shelton, CT, USA) and Qubit 2.0 (Thermo Fisher Scientific, Waltham, MA, USA All 24 RNA samples had RIN values between 7 and 9.2 and were thus appropriate for library preparation, which was conducted by Flinders University Genomics Facility. The TruSeq stranded mRNA library prep kit (Illumina) was used to create each library starting with between 200 ng and 1 μg RNA as per standard protocol. Pooled equimolar libraries were quality-checked and sequenced at the Deakin University Genomics Centre on a NovaSeq S4 flow cell, with paired-end 2 × 150 bp, to achieve an average coverage of 30 million reads per sample. Raw data were deposited at NCBI as BioProject ID PRJNA1069118. 

#### 5.3.2. RNA-Seq Read Quality Control

Transcriptome analysis was conducted at Queensland University of Technology (QUT), Australia. Raw sequence data were quality-control-checked using FastQC v0.11.9 [73] (Figure S7). Paired-end reads were trimmed to ensure data quality using Trimmomatic v.0.36 [74]. Illumina’s TruSeq adapter sequences were removed, and parameters otherwise mimicked those used by the Trinity de novo assembler [75], i.e., “ILLUMINACLIP:${ADAPTERS}8:2:30:10 SLIDINGWINDOW:4:5 LEADING:5 TRAILING:5 MINLEN:25”. Resulting quality-trimmed reads were used for all downstream analyses.

#### 5.3.3. De Novo Transcriptome Assembly

All 24 transcriptome samples, regardless of treatment, were de novo assembled to create a global transcriptome library for *E. quadricolor*. Our process for creating a high-quality transcriptome assembly made use of several transcriptome assemblers, specifically, SOAPdenovo-Trans v.1.03 [76] and Oases v.0.2.09 [77] assemblers were used to build transcriptomes with several k-mer lengths (23, 25, 31, 39, 47, 55 and 63 for both and 71 additionally for SOAPdenovo-Trans only). Trinity v.2.14.0 [75] was also used with parameters, including min_kmer_cov = 2 and SS_lib_type = RF. All resulting transcriptome files were concatenated and sequences shorter than 250 bp were removed to eliminate potentially poor quality and/or fragmented transcripts.

The concatenated file was subjected to the EvidentialGene v.2022.01.20 tr2aacds pipeline using ≥30 amino acids as the ORF cut-off [78]. This process is designed to receive a massively redundant transcriptome from multi-k-mer assembly and produce a non-redundant output containing the best-assembled transcripts from each assembler; it additionally predicts coding regions within these transcripts. The resultant transcriptome was assessed for quality using BUSCO v.5.2.1 [79].

#### 5.3.4. Contaminant Removal

PsyTrans [34] was used to remove transcripts arising from endogenous symbionts. PsyTrans is a script which utilises protein sequences from sea anemone species related to *E. quadricolor* as well as protein sequences from symbionts to identify and remove contaminants from the transcriptome.

We created a custom database of symbiont sequences using published genomic and transcriptomics resources for Symbiodinium and related organisms [80,81,82,83] (Table S4). When a data source only provided nucleotide transcripts, we used TransDecoder v.5.6.0 [84] to obtain translated coding DNA sequence predictions. We also opted to use solely genomic resources for related sea anemone species, avoiding transcriptomics data which may itself contain symbiont contaminants. Genomic data were sourced from other members of the family Actiniidae, namely *Actinia equina* [85], *A. tenebrosa* [5] (GenBank GCA_029948245.1) and *Aulactinia veratra* [36] (Table S5).

Sequences from related sea anemone species and from symbionts had their redundancy reduced through use of CD-HIT v.4.6 [86] with parameters (-c 0.95 -n 5 -aS 0.9). The resulting files were provided to PsyTrans with default parameters; the output FASTA file corresponding to predicted sea anemone host transcripts represented our initial de novo transcriptome (Supplementary File S1).

#### 5.3.5. Clustering and Read Counting

To obtain read counts associated with the gene level rather than individual transcripts, we first used salmon v.1.9.0 [87] with parameters (--libType A --dumpEq --hardFilter --skipQuant) to produce equivalence classes for the reads from each sample against the transcriptome file. Following this, Corset v.1.09 [88] used the salmon equivalence classes to cluster transcripts based on shared read alignments and expression patterns and provided normalised read counts associated with each cluster (which putatively represents a gene). Gene clusters were used for gene annotation and normalised read counts were used to determine gene cluster abundance, as described below in Section 5.3.9.

#### 5.3.6. Gene Annotation and Functional Enrichment Analysis

Predicted coding DNA sequences (translated proteins) from our transcriptome (Supplementary File S1) were queried against the UniRef90 database [89] using MMseqs2 v.fcf5260 [90]. Gene names for queried sequences were attributed based on their best match, and functional annotation of gene ontology (GO) [91,92] was performed by identifying the best match which had GO annotations in UniProt’s idmapping_selected.tab file. Annotated GO terms were expanded to include ancestor terms using the Python library goatools [93]. Final annotation of the 279,274 tentacle gene clusters is found in Supplementary File S3.

#### 5.3.7. Toxin Annotation Pipeline

Toxin annotation was accomplished using custom scripts available from https://github.com/zkstewart/Various_scripts/tree/master/Toxins_annot. As an overview, this process leverages custom-made hidden Markov models (HMMs) and the HMMer software [94] to predict protein domains located in venom protein sequences. Python scripts assess the results of HMMer searches to determine whether a sequence is likely to be part of a sea anemone associated toxin family. The custom-made HMMs were generated from an initial dataset of six host and non-hosting sea anemone venom proteomes i.e., *A. tenebrosa*, *Aiptasia pulchella*, *Heteractis malu*, *Macrodactyla doreensis*, *Telmatactis* sp. and *S. haddoni* [36]. Multiple sequence alignments (MSAs) of venom families were formed using a mixture of manual inspection of sequences (with an emphasis on visually locating conserved regions likely to be important to protein structure, e.g., cysteine residue organisation), assisted by BLASTp [95,96] and HMMer searches to find sequence identity. Importantly, this process focused on gene families present in two or more of the sea anemone venom proteomes; genes found in only one species were excluded from further consideration even if they were known to be venom toxins from previous studies. MSAs were manually trimmed to adjust the domain regions from within family alignments and were converted into HMMs. Scripts were created by manually tuning a rule-based process which considers the HMMer results obtained for each sequence, including the domains which hit against a sequence and an E-value ≤ 1 × 10^−5^ as the cut-off for significance, in addition to considering sequence features, including the relative positions of the domain hits in a sequence. Through this process, many previously discovered toxin families were modelled and made easily predictable using this system. Additionally, several toxin families were identified which have not been reported on previously (U# and Z# models) and hence have unknown functionality [36].

Toxins from the pipeline that were assigned as uncharacterised toxins were manually inspected for toxin domains that could be assigned to a venom category using ScanProsite [97]. In addition, toxin families identified by Delgado et al. [29] as present in the *E. quadricolor* transcriptome but not found through our pipeline were manually added if the appropriate domain was found using ScanProsite (Supplementary File S3).

#### 5.3.8. Signal P

SignalP v.5.0b [98] was used to predict signal peptides in protein sequences using default settings, i.e., using Eukarya prediction.

#### 5.3.9. Normalised Abundance of Transcripts

DESeq2-normalised counts [35], which uses a ‘median of ratios’ methodology, was used to normalise transcript expression data across our 24 tentacle RNA-seq samples and produce transcript abundance data for each gene cluster. These transcript counts were averaged across the 24 RNA-seq samples and then summed for all gene clusters assigned to a toxin protein family to obtain gene expression levels for that protein family. Normalised counts were used over TMP or FPKM in this study as normalised count data have been shown to have lower coefficient of variation (CV) and higher interclass correlation (ICC), particularly as we are comparing across samples (*n* = 24) and individuals *(n* = 6) [99,100].

### 5.4. Proteomics

To identify all proteins in the milked venom, data-dependent acquisition (DDA) analysis with gas fractionation using an Orbitrap Fusion™ Lumos™ Tribrid™ Mass Spectrometer (Thermo Fisher Scientific, Waltham, MA, USA) was conducted on a pooled venom sample to create a spectral library of all proteins.

#### 5.4.1. Venom Protein Extraction for Mass Spectrometry

Lyophilised venom from four *E. quadricolor* individuals in the non-hosting period and four *E. quadricolor* individuals in the hosting period underwent proteomics analysis at the Flinders University Omics Facility. A 50 µg protein pool of all eight samples (25 µg non-hosting:25 µg hosting) was reduced and alkylated following a standard procedure. Briefly, a mixture of hydrophobic and hydrophilic Sera-Mag Carboxylate SpeedBeads (Cytiva, Marlborough, MA, USA) was used for protein clean-up and trypsin digestion following the manufacturer’s instructions. Following the trypsin digestion, peptides in each sample were cleaned up with a 200 µL C18 StageTip (Thermo Fisher Scientific, Waltham, MA, USA) and eluted in 80% acetonitrile/0.1% formic acid. The sample was then dried down in a Christ RVC 2-25 CD plus vacuum concentrator (Christ, Osterode am Harz, Germany) and resuspended in 5% acetonitrile. Approximately 5.8 µg peptides were recovered from the pooled venom sample as measured by the NanoDrop Spectrophotometer (Thermo Fisher Scientific, Waltham, MA, USA).

#### 5.4.2. Spectral Library Creation via DDA and GPF

DDA analysis used an Orbitrap Fusion™ Lumos™ Tribrid™ Mass Spectrometer (Thermo Fisher Scientific, Waltham, MA, USA) equipped with a Nanospray Flex™ Ion Source (ES071, Thermo Fisher Scientific) coupled to a Dionex Ultimate 3000 UPLC chromatography system (Thermo Fisher Scientific). Pooled venom tryptic peptides (4.8 µg) were injected into a PepMap™ 100 trap column (0.3 × 5 mm, 5 µm C18, Thermo Fischer) and then eluted onto an inhouse pulled column created from a 75 µm inner diameter fused silica capillary packed with 3 µm ReproSil-Pur C18 beads (Dr. Maisch, Ammerbuch, Germany) to a length of 15 cm. The column was heated to 60 °C using a Nanospray Flex™ Column Oven (Sonation lab solutions, Biberach, Germany) and the flow rate for the gradient pump was 300 nL per minute. The column and trap were equilibrated in Solvent A (0.1% formic acid in water) and eluted with solvent B (79.9% acetonitrile, 20% water, 0.1% formic acid) using a 2–30% linear gradient over 55 min (Table 5). Total run time was 85 min and internal mass calibration using RunStart EASY-IC™ (Thermo Fisher Scientific, Waltham, MA, USA) was enabled.

Gas phase fractionation was employed in conjunction with DDA for this analysis (methods 2–7, Table 6). Gas phase fractionation (GPF) separates peptides in the gas phase; i.e., once peptides have entered the instrument in the gas phase, peptides in each phase are run through six identical methods following the DDA protocol. It is implemented by analysing multiple injections of the same sample with 50–400 *m*/*z* mass windows analysed in each injection. With only the MS scan range changing for each method, the *m*/*z* range overlapped by 10 da between methods to ensure no peptides were missed (Table 6).

All DDA files collected were used to generate a spectral library using Spectronaut software V16.022 with default settings (Biognosys AG, Schlieren, Switzerland). The spectral library was searched against the predicted protein sequences from the assembled *E. quadricolor* transcriptome (Supplementary File S1) to identify proteins present in the venom proteome.

#### 5.4.3. Normalised Abundance of Protein Expression Utilising DIA

The spectral library was used as a database to perform data-independent analysis (DIA) mass spectrometry on individual venom samples. Lyophilised venom from seven *E. quadricolor* individuals underwent proteomics analysis at the Flinders University Omics Facility. Following the same methods above, 10 µg protein from each individual venom sample was reduced and alkylated, and approximately 0.9–1.5 µg of peptides were recovered from each of the seven samples as measured by a NanoDrop. For measuring protein expression levels, approximately 1 µg of tryptic peptides (6.4 µL) from each individual sample was injected into a PepMap™ 100 trap column (0.3 × 5 mm, 5 µm C18, Thermo Fisher Scientific, Waltham, MA, USA) using the methods described in 5.4.2. For DIA runs, the Thermo Fusion Lumos was configured to acquire 34 variable *m*/*z* precursor isolation windows spanning 350–1200 *m*/*z* mass range, with a 1 *m*/*z* overlap between windows. Precursor spectra over a 350–1200 *m*/*z* mass range were acquired prior to DIA scans employing RunStart EASY-ICTM (Thermo Fisher Scientific, Waltham, MA, USA) as the internal mass calibration with a resolution of 120,000, and AGC target 8 × 10^−5^ and dynamic maximum inject time were used for all full scan MS spectra. An ms2 resolution of 30,000, AGC target 1 × 10^−6^, dynamic maximum inject time mode, and normalised fixed HDC collision energy of 30% were employed for all DIA scans. Spectronaut software V16.022 (Biognosys AG, Schlieren, Switzerland) using factory (default) settings was used for peak detection, deconvolution and normalisation of the spectra utilising the spectral library and to determine levels of the proteins detected in each venom sample. Briefly, quantification was performed at the ms^2^ level (ms/ms) with fragment ions that passed filtering used for quantification that was calculated by measuring the area under the curve between the XIC peak boundaries for each target ion.

### 5.5. Data Analysis

The venom profiles were visualised using PieDonut from the ‘webr’ R package [101]. Bar and pie graphs were created in Excel.

## Figures and Tables

**Table 1:** Summary of tentacle transcriptome and venom proteome gene clusters found in *Entacmaea quadricolor*. ORFs = open reading frames.

	RNA Gene Clusters	Protein Gene Clusters
ORF sequences	279,274	5375
Annotated ORF sequences	72,218	5213
ORFs with GO terms	46,288	3901
Unique UniRef90 Hits	18,469	3718
ORFs with signal sequence	11,807	1224
Putative toxin ORFs	1251	230
Putative toxin ORFs with signal sequence	515	149
Putative toxin unique UniRef90 Hits	296	124

**Table 2:** Percentage of *E. quadricolor* toxin gene clusters detected in transcriptome and proteome data by venom category. TF = number of different toxin protein families represented in each venom category.

Venom Category	RNA Gene Clusters	TF	Protein Gene Clusters	TF	Percentage (%) ^1^
Allergen and innate immunity	9	3	4	2	44.4
Auxiliary	67	1	19	1	28.4
Haemostatic and haemorrhagic	409	5	55	5	13.4
Mixed function enzymes	24	1	1	1	4.2
Neurotoxins	122	9	21	5	17.2
Pore forming	45	5	16	4	35.6
Protease inhibitors	38	2	5	1	13.2
Unknown	537	16	109	11	20.3

^1^ Percentage equals number of different gene clusters identified in the venom proteins divided by the total number of gene clusters identified in the de novo transcriptome in each venom category.

**Table 3:** Comparison of the top 10 toxin protein families based on number of gene clusters in (**a**) tentacle transcriptome and (**b**) venom proteome. Protein families in the top 10 at both transcript and protein level are highlighted light grey. GC = gene cluster.

(a) Venom Category	Toxin Protein Family	RNA Gene Clusters	Rank of GC in Proteome
Haemostatic and haemorrhagic	Coagulation factor V-like	315	2
Unknown	IG-like	300	1
Unknown	Uncharacterised toxins	104	3
Neurotoxin	ShK-like	85	5
Auxiliary	Peptidase M12A	67	4
Haemostatic and haemorrhagic	Peptidase S1	37	3
Haemostatic and haemorrhagic	Ficolin lectin family	30	10
Unknown	EGF-like	25	11
Mixed function enzymes	PLA2	24	13
Pore Forming	Actinoporins	23	7
**(b) Venom Category**	**Toxin Protein Family**	**Protein Gene** **Clusters**	**Rank of GC in** **Transcriptome**
Unknown	IG-like	56	2
Haemostatic and haemorrhagic	Coagulation factor V-like	22	1
Haemostatic and haemorrhagic	Peptidase S1	21	6
Unknown	Uncharacterised toxins	21	3
Auxiliary	Peptidase M12A	19	5
Neurotoxin	ShK-like	14	4
Unknown	U15	11	11
Pore forming	Actinoporins	7	10
Haemostatic and haemorrhagic	Peptidase M12B	6	12
Unknown	Z3	6	13
Unknown	U12	6	17
Pore forming	DELTA-alicitoxin-Pse2b-like	5	18
Protease inhibitor	Venom Kunitz-type family	5	15
Haemostatic and haemorrhagic	Ficolin lectin family	4	7

**Table 4:** Comparison of the top 10 most abundant toxin protein families in (**a**) tentacle transcriptome and (**b**) venom proteome. TF = toxin protein family. Protein families in the top 10 at both transcript and protein level are highlighted light grey.

(a) Venom Category	Toxin Protein Family	RNA Normalised Abundance ^1^	Rank of TF in Proteome
Unknown	Z3	56,348	1
Unknown	Sea Anemone 8	54,210	Not present
Neurotoxin	ShK-like	48,215	18
Unknown	IG-like	34,585	7
Unknown	Uncharacterised toxins	24,521	16
Unknown	EGF-like	19,030	5
Mixed function enzyme	PLA2	11,305	4
Auxiliary	Peptidase M12A	8987	13
Haemostatic and haemorrhagic	Factor V-like	8767	19
Unknown	CREC	6308	15
**(b) Venom Category**	**Toxin Protein Family**	**Protein Normalised** **Abundance ^2^**	**Rank of TF in** **Transcriptome**
Unknown	Z3	16,933,968	1
Haemostatic and haemorrhagic	Peptidase S1	9,294,230	11
Protease inhibitor	Venom Kunitz	6,409,895	13
Mixed function enzyme	PLA2	4,873,099	7
Unknown	EGF-like	4,773,700	6
Unknown	U15	4,298,257	27
Unknown	IG-like	3,884,603	4
Unknown	U12	1,416,547	17
Allergen and innate immunity	CAP	1,367,399	14
Neurotoxin	NEP 3 family	911,820	16

All toxin gene clusters were assigned to a toxin protein family and a venom category. ^1^ Sum of RNA transcript average abundance across gene clusters in the toxin protein family from *n* = 24 RNA samples. ^2^ Sum of protein average abundance across gene clusters in the toxin protein family from *n* = 7 venom samples.

**Table 5:** HPLC chromatography gradient.

Time	Solvent B
0 min	2%
5 min	2%
10 min	8%
60 min	31.2%
66 min	50%
69 min	100%
72 min	100%
75 min	2%

**Table 6:** *m*/*z* scan ranges used in each method performed on each GPF fraction.

Method	*m*/*z*
1	350–1200
2	350–500
3	490–550
4	540–610
5	600–710
6	700–810
7	800–1200

## Data Availability

Raw RNA seq transcript data have been uploaded to NCBI BioProject ID PRJNA1069118 (https://www.ncbi.nlm.nih.gov/sra/PRJNA1069118). All proteomics raw files, Spectronaut sne files, Spectronaut reports, FASTA files and the experimental template have been uploaded to PRIDE(35) under accession number PXD048736 (http://www.ebi.ac.uk/pride/archive/projects/PXD048736).

## Supplementary Materials

The following supporting information can be downloaded at: https://www.mdpi.com/article/10.3390/toxins16020085/s1. Table S1. Putative toxins present in *Entacmaea quadricolor;* Table S2. The architecture of the 56 IG-like gene clusters detected in *E. quadricolor* venom proteomics data; Table S3. Toxin family domain architecture of 21 putative toxin proteins assigned to the unknown venom category detected in venom; Table S4. Symbiont data sources; Table S5. Anemone data sources; Figure S1. Multiple sequence alignment of *E. quadricolor* IG-like family proteins; Figure S2. Multiple sequence alignment of Peptidase S1 toxins identified in milked *E. quadricolor* venom; Figure S3. Multiple sequence alignment of Kunitz-type family toxins identified in milked *E. quadricolor* venom with other Kunitz-type proteins; Figure S4. Multiple sequence alignment of the secreted PLA2 detected in *E. quadricolor* venom with other homologous snake and sea anemone PLA2 proteins; Figure S5. Alignment of *E. quadricolor* proteins present in venom identified as toxins targeting Type I Kv channels; Figure S6. Multiple sequence alignment of the actinoporins detected in *E. quadricolor* venom with homologous actinoporins from host and non-host sea anemones; Figure S7. Quality control graphs for RNA sequencing; Supplementary File S1. cassie_okay-okalt.proteomics.aa; Supplementary File S2. File S2 Proteome Final Library.csv; Supplementary File S3. File S3 Transcriptome Final Library.csv. references in Supplementary Materials [102,103].
