# Supplementary material for: Proteotransciptomics of the Most Popular Host Sea Anemone Entacmaea quadricolor Reveals Not All Toxin Genes Expressed by Tentacles Are Recruited into Its Venom Arsenal
Source: Toxins (Basel). 2024 Feb 5;16(2):85. doi: 10.3390/toxins16020085 (PMC10893224; doi:10.3390/toxins16020085)

### Table S1: Putative toxins present in *Entacmaea quadricolor*. % indicates percentage of gene clusters in the tentacle transcriptome that were found in the venom proteome. * indicates toxin families that were manually identified. / no specific protein domain identified.

| Toxin Family ID | Domain | Tentacle transcriptome | Venom proteome |  |  |
| --- | --- | --- | --- | --- | --- |
|  |  | **Gene Clusters** | **Proteins** | **%** |  |
| ALLERGEN AND INNATE IMMUNITY |  | **9** | **4** | **44.4** |  |
| CRISP | CAP | 7 | 3 | 28.6 | * |
| DPP IV/FAP | Peptidase S9 | 1 | 1 | 100 | * |
| HYAs | / | 1 |  | 0 | * |
| AUXILIARY |  | **67** | **19** | **28.4** |  |
| Peptidase M12A | Astacin | 67 | 19 | 28.4 |  |
| HAEMOSTATIC AND HAEMORRHAGIC |  | **409** | **55** | **13.5** |  |
| True venom lectin family | C-type lectin | 7 | 2 | 28.6 | * |
| Coagulation factor V-like | F5/8 type-C | 315 | 22 | 7 |  |
| Ficolin lectin family | Fibrinogen  C-terminal | 30 | 4 | 13.3 | * |
| Peptidase M12B | TSP type-1 | 20 | 6 | 30 | * |
| Peptidase S1 | Trypsin | 37 | 21 | 56.8 |  |
| MIXED FUNCTION ENZYMES |  | **24** | **1** | **4.2** |  |
| Phospholipase A2 | PLA2 | 24 | 1 | 4.2 |  |
| NEUROTOXIN |  | **122** | **21** | **17.2** |  |
| Acrorhagin | / | 3 |  | 0 |  |
| Delta-actitoxin-Eqd1a | ATX- III | 2 |  | 0 | * |
| BßH-like (Type IV Kv channel) | / | 4 | 1 | 25 |  |
| ß-Defensin (Type III Kv channel) | Defensin | 2 |  | 0 |  |
| CRISP | CAP/ShKT | 2 | 2 | 100 | * |
| ICK-like (Type V Kv channel) | / | 17 |  | 0 |  |
| NEP 3 Family | ShKT | 5 | 2 | 40 | * |
| SCRiP (TRPA1) | / | 2 |  | 0 |  |
| ShK-like (Type I Kv channel) | ShKT | 85 | 14 | 16.5 |  |
| PORE FORMING |  | **45** | **16** | 35.6 |  |
| Actinoporin (Type II cytolysins) | Cytolysin | 23 | 7 | 30.4 |  |
| DELTA-actitoxin-Ucs1a | Cytolysin | 9 | 2 | 22.2 | * |
| \| DELTA-alicitoxin-Pse2a-like \| \| --- \| | MAC/PF | 2 | 2 | 100 | * |
| \| DELTA-alicitoxin-Pse2b-like \| \| --- \| | MAC/PF | 10 | 5 | 50 | * |
| DELTA-thalatoxin-Avl2a-like | MAC/PF | 1 |  | 0 | * |
| PROTEASE INHIBITOR |  | **38** | **5** | **13.2** |  |
| Venom Kunitz-type family | Kunitz-BPTI | 19 | 5 | 26.3 |  |
| Kazal-like | Kazal | 19 | 0 | 0 |  |

| Toxin Family ID | Domain | Tenacle transcriptome | Venom Proteome |  |  |
| --- | --- | --- | --- | --- | --- |
|  |  | **Gene Clusters** | **Proteins** | **%** |  |
| UNKNOWN |  | **537** | **109** | **20.3** |  |
| CREC | EF-hand | 8 | 2 | 25 |  |
| EGF-like | EGF-like | 25 | 3 | 12 |  |
| Immunoglobulin-like | IGC2-like | 300 | 56 | 18.7 |  |
| Lipase maturation factor | LMF1 | 8 |  | 0 |  |
| Sea anemone 8 toxin family | / | 10 |  | 0 |  |
| U2 |  | 9 |  | 0 |  |
| U8 |  | 5 |  | 0 |  |
| U9 |  | 1 |  | 0 |  |
| U11 |  | 4 |  | 0 |  |
| U12 | Folate receptor | 13 | 6 | 46.2 |  |
| U13 | Folate receptor | 4 | 1 | 25 |  |
| U15 |  | 21 | 11 | 52.4 |  |
| U16 |  | 4 | 1 | 25 |  |
| Z3 | Zona pellucida | 20 | 6 | 30 |  |
| Z7 | Zona pellucida | 1 | 1 | 100 |  |
| uncharacterised toxins | / | 104 | 21 | 20.2 |  |
| Total |  | **1,251** | **230** | **18.4** |  |

**Table S2: The architecture of the 56 IG-like gene clusters detected in *E. quadricolo*r venom proteomics data.** Protein domains were predicted using the ScanProsite tool [37].

|  | **CLUSTER No** | **Schematic of the Scan Prosite Architecture** | **No of AA** | **No of IG-like Domains** |
| --- | --- | --- | --- | --- |
| 1 | 31550.104775 | 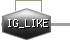 | 115 | 1 |
| 2 | 31550.46484 | 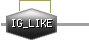 | 147 | 1 |
| 3 | 31550.105257 | 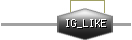 | 218 | 1 |
| 4 | 31550.97200 | 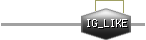 | 241 | 1 |
| 5 | 31550.94511 | 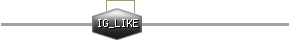 | 481 | 1 |
| 6 | 31550.152689 | 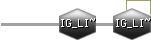 | 250 | 2 |
| 7 | 31550.46477 | 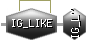 | 143 | 2 |
| 8 | 31550.116110 | 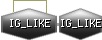 | 169 | 2 |
| 9 | 31550.46481 | 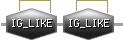 | 204 | 2 |
| 10 | 31550.81860 | 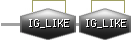 | 216 | 2 |
| 11 | 31550.94434 | 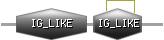 | 271 | 2 |
| 12 | 31550.136340 | 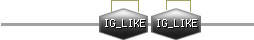 | 422 | 2 |
| 13 | 31550.86496 | 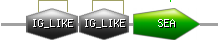 | 363 | 2 |
| 14 | 31550.80682 | 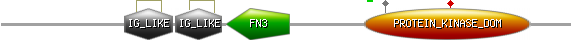 | 951 | 2 |
| 15 | 31550.84494 | 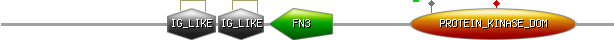 | 1022 | 2 |
| 16 | 31550.115879 | 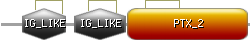 | 416 |  |
| 17 | 31550.27744 | 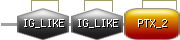 | 298 | 2 |
| 18 | 31550.51670 | 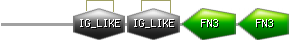 | 481 | 2 |
| 19 | 31550.107372 | 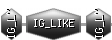 | 185 | 3 |
| 20 | 31550.125505 | 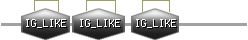 | 411 | 3 |
| 21 | 31550.138535 | 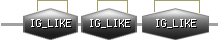 | 362 | 3 |
| 22 | 31550.138697 | 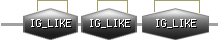 | 362 | 3 |
| 23 | 31550.86443 | 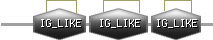 | 354 | 3 |
| 24 | 31550.68053 | 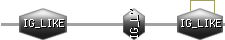 | 374 | 3 |
| 25 | 31550.120766 | 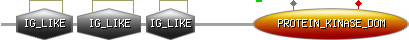 | 680 | 3 |
| 26 | 31550.83000 | 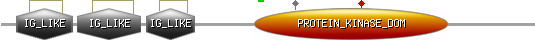 | 890 | 3 |
| 27 | 31550.133646 | 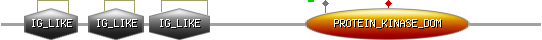 | 901 | 3 |
| 28 | 31550.93983 | 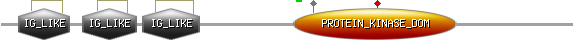 | 954 | 3 |
| 29 | 31550.123076 | 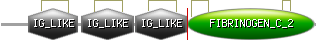 | 525 | 3 |
| 30 | 31550.83723 | 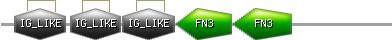 | 652 | 3 |
| 31 | 31550.112712 | 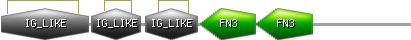 | 684 | 3 |
| 32 | 31550.76013 | 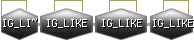 | 321 | 4 |
| 33 | 31550.86456 | 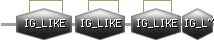 | 355 | 4 |
| 34 | 31550.86457 | 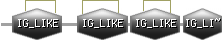 | 369 | 4 |
| 35 | 31550.100393 | 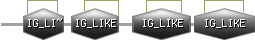 | 423 | 4 |
| 36 | 31550.81859 | 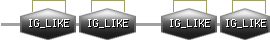 | 449 | 4 |
| 37 | 31550.86464 | 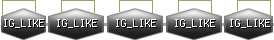 | 455 | 5 |
| 38 | 31550.41424 | 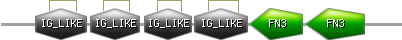 | 669 | 4 |
| 39 | 31550.106382 | 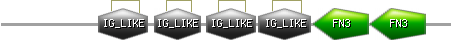 | 751 | 4 |
| 40 | 31550.150871 | 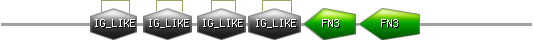 | 886 | 4 |
| 41 | 31550.137363 | 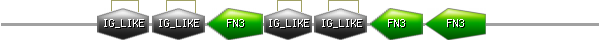 | 997 | 4 |
| 42 | 31550.85420 | 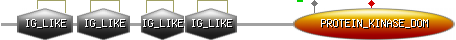 | 758 | 4 |
| 43 | 31550.86448 | 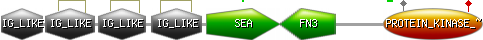 | 804 | 4 |
| 44 | 31550.76001 | 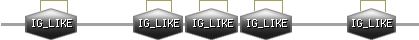 | 697 | 5 |
| 45 | 31550.93534 | 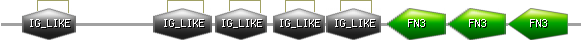 | 967 | 5 |
| 46 | 31550.109742 | 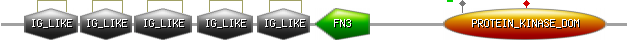 | 1044 | 5 |
| 47 | 31550.107245 | 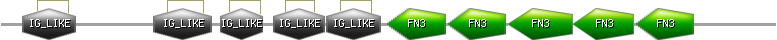 | 1293 | 5 |
| 48 | 31550.84836 | 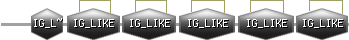 | 580 | 6 |
| 49 | 1550-82983 | 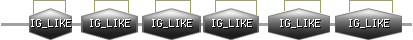 | 686 | 6 |
| 50 | 31550.76010 | 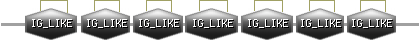 | 697 | 7 |
| 51 | 31550.122563 | 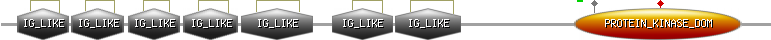 | 1284 | 7 |
| 52 | 31550.121071 | 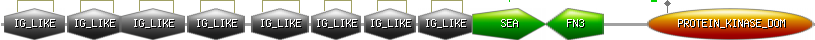 | 1357 | 8 |
| 53 | 31550.137307 | 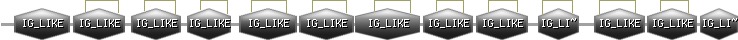 | 713 | 13 |
| 54 | 31550.130489 | 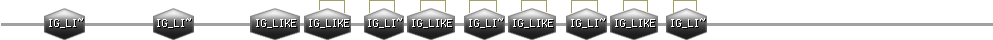 | 1654 | 11 |
| 55 | 31550.75997 | 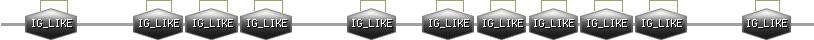 | 1355 | 11 |
| 56 | 31550.133837 | 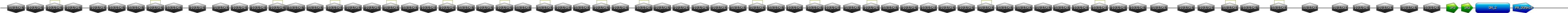 | 8067 | 71 |

Table S3. Toxin family domain architecture of 21 putative toxin proteins assigned to the unknown venom category detected in venom. Protein domains were predicted using the ScanProsite tool [37].

| **CLUSTER No** | **Schematic of the Scan Prosite Architecture** | **No of AA** | **SHK**  **domain** |
| --- | --- | --- | --- |
| 31550.118416 | No SHK-like domain predicted by ScanProsite result  Match back to U-actitoxin Aer2a which is contains SH-like domain missing 2 cysteines | 87 | 0 |
| 31550-55117 | No SHK-like domain predicted by ScanProsite result  Match back to U-actitoxin Aer2a which is contains SH-like domain missing 2 cysteines | 87 | 0 |
| 67336.1 | No SHK-like domain predicted by ScanProsite result but all cysteines for a SHK-like domain present | 121 | 0 |
| 31550.33431 | 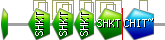 | 274 | 5 |
| 31550.51403 | 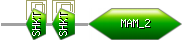 | 302 | 2 |
| 31550.42521 | 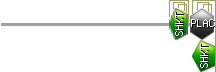 | 358 | 2 |
| 31550.177018 | 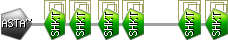 | 379 | 6 |
| 31550.34598 | No domains, no SHK like cysteines, matches a insecticidal delta-endotoxin Cry8Ea1 | 456 | 0 |
| 31550.6213 | 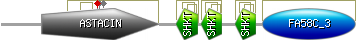 | 593 | 3 |
| 31550.133892 | 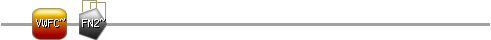 | 816 | 0 |
| 31550.144559 | 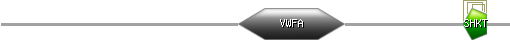 | 849 | 1 |
| 31550.129577 | 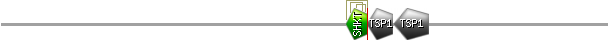 | 1012 | 1 |
| 31550.117316 | 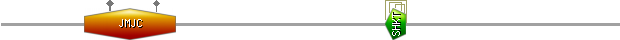 | 1032 | 1 |
| 31550.129509 | 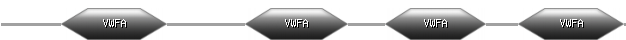 | 1042 | 0 |
| 31550.126088 | 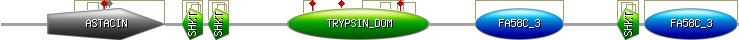 | 1230 | 3 |
| 31550.101868 | 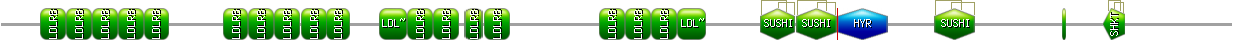 | 2053 | 1 |
| 31550.98207 | 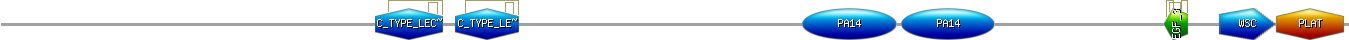 | 2247 | 0 |
| 31550.146039 | 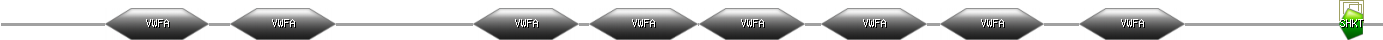 | 2304 | 1 |
| 31550.50366 | 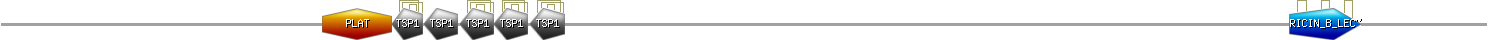 | 2478 | 0 |
| 31550.87127 | 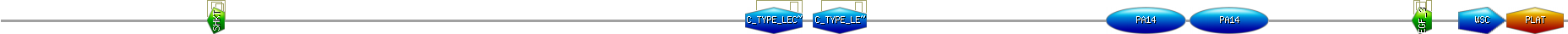 | 3092 | 2 |
| 31550.46237 | 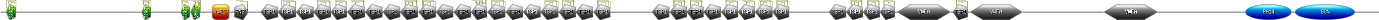 | 4637 | 4 |

### Table S4 :Symbiont data sources

| Citation | Species |
| --- | --- |
| González-Pech et al.,2021 [82] | - *S. linucheae* (genome) - *S. microadriaticum CassKB8* (genome) - *S. microadriaticum 04-503SCI.03* (genome) - *S. natans* (genome) - *S. necroappetens* (genome) - *S. tridacnidorum* (genome) |
| Levin et al., 2016[83] | - Mixed *Symbiodinium* population isolated from *Acropora tenuis* from South Molle Island (transcriptome) - Mixed *Symbiodinium* population isolated from *Acropora tenuis* from Magnetic Island (transcriptome) |
| Camp et al., 2022 [81] | - *Cladocopium goreaui* (transcriptome) - *Durusdinium trenchii* (transcriptome) - *Breviolum* sp. (transcriptome) |
| Arriola et al., 2018 [80] | - *Micractinium conductrix* (genome) - *Chlorella sorokiniana* UTEX 1602 (genome) |

### Table S5: Anemone data sources

| Citation | Species |
| --- | --- |
| Wilding et al., 2020 [85] | - *Actinia equina* (genome) |
| Ashwood et al. 2023 [5] | - *Actinia tenebrosa* (genome) |
| (Unpublished, ePGL) | - *Aulactinia veratra* (genome) |


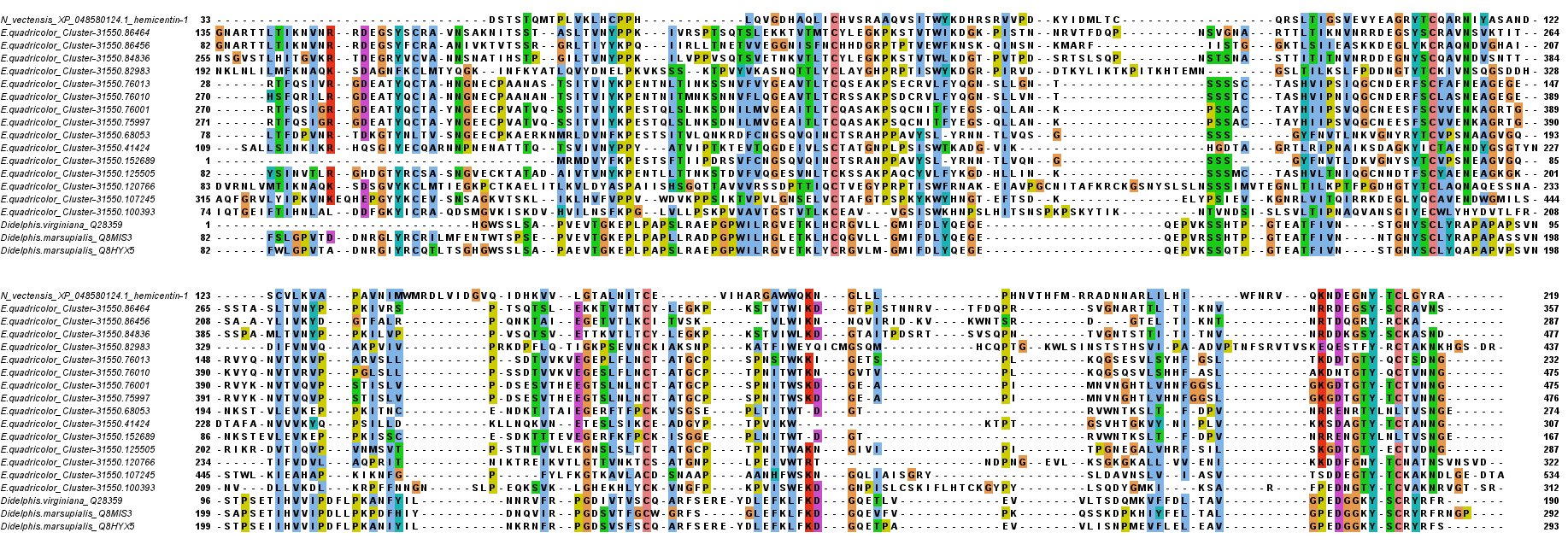


**Figure S1: Multiple sequence alignment of *E. quadricolor* IG-like family proteins present in venom.** Conserved cysteine residues are indicated with the blue star symbol. IG-like family conserved regions marked with a black box. Alignment was created using MafftWS alignment algorithm and visualised in Jalview with the Clustal colour scheme[102]. Accession numbers for the homologous venom inhibitor proteins from *Didelphis marsupiali* (DM43 and DM64) and *D. virginiana* (Alpha 1B-glycoprotein) are listed in the alignment.


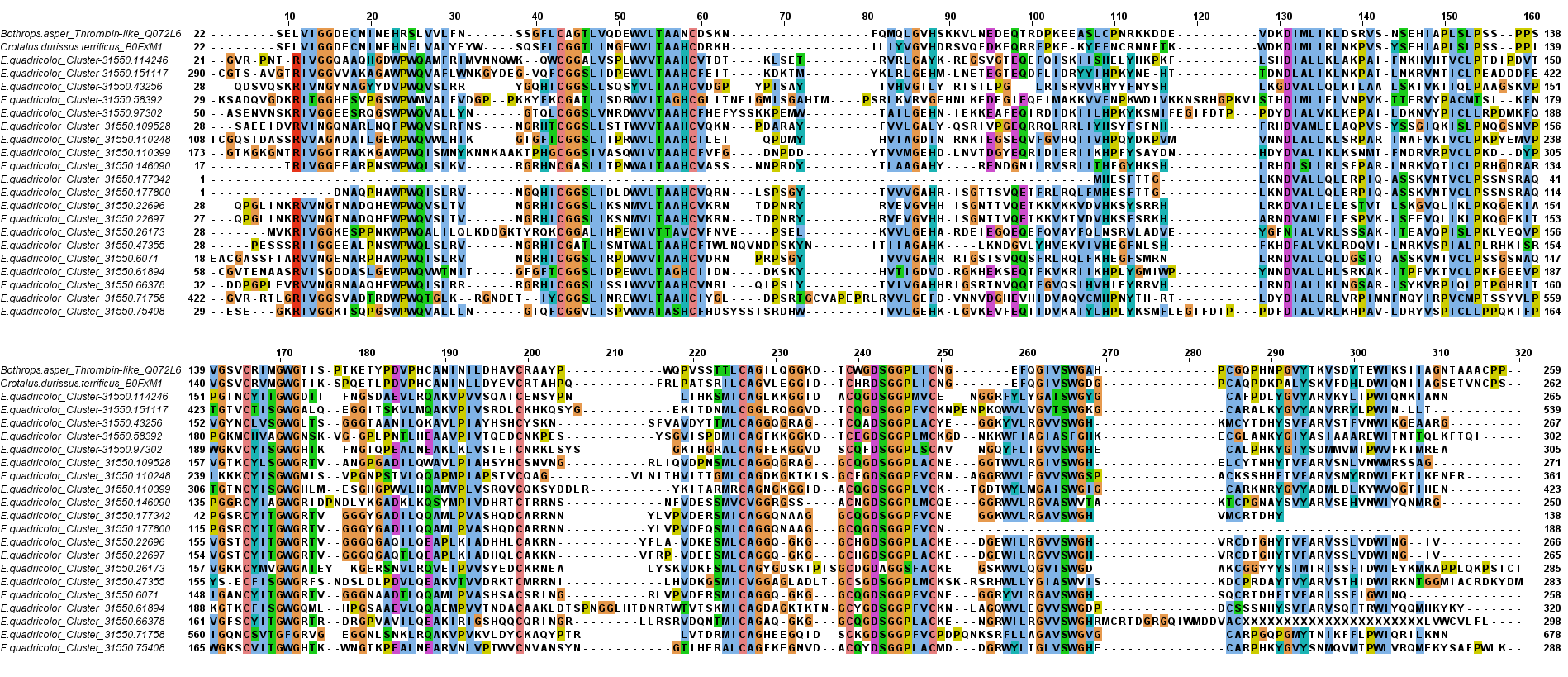


**Figure S2: Multiple sequence alignment of Peptidase S1 toxins identified in milked *E. quadricolor* venom.** Alignment was created and visualised using ClustalWS alignment algorithm in Jalview [101]. All but one *E. quadricolo*r gene cluster (*Cluster-31550.26173)* encodes a trypsin-like serine protease protein with the conserved His, Asp and Ser residues of the trypsin domain indicated with a black box and arrow . Conserved cysteine residues are indicated with the blue star symbol. Accession numbers for Peptidase S1 toxins, asperase from *Bothrops aspe*r (pit viper) and gyroxin from *Crotalus durissus terrificus* (South American rattlesnake) proteins are indicated in the alignment.


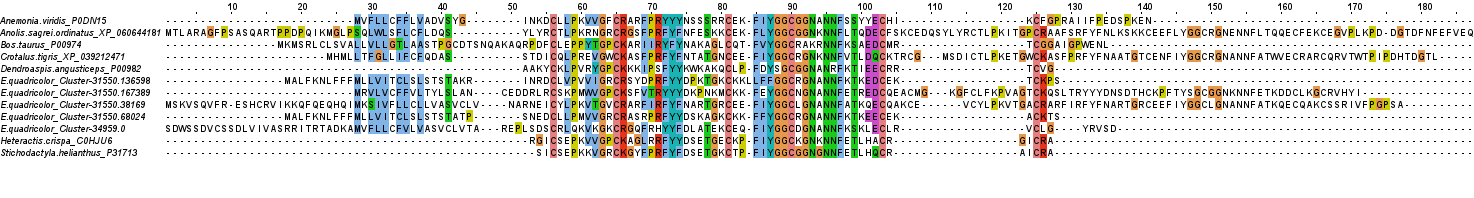


Key residues required for trypsin inhibition: G KA I GG R


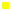


**Figure S3: Multiple sequence alignment of Kunitz-type family toxins identified in milked *E. quadricolor* venom with other Kunitz-type proteins.** Alignment was created and visualised using ClustalWS alignment algorithm in Jalview [101]. The six conserved cysteine residues that form the prototype signal for a pancreatic trypsin inhibitor are indicated with the symbol. Indicates other conserved residues that have been identified as important for trypsin inhibition [41]. Alignment also includes Kunitz type family members from anemones, snakes and BPTI a *Bos taurus* trypsin inhibitor. Accession numbers are indicated in the alignment.


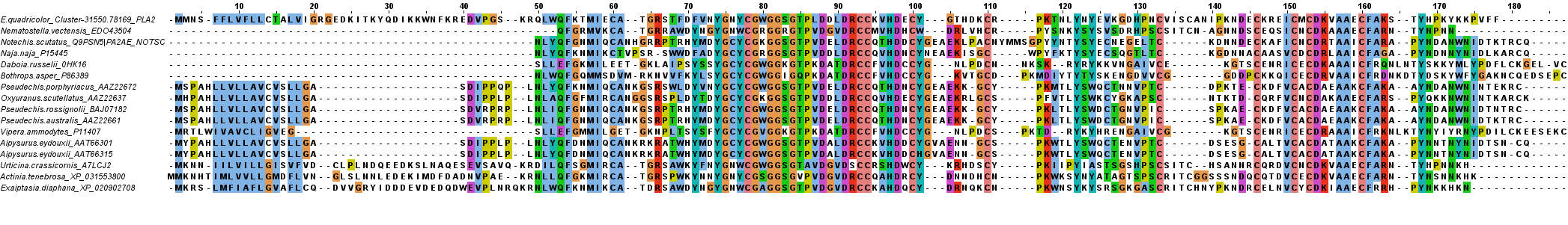


Ca2+ loop Active site His, Asp, Tyr

C-terminal extension

prepeptide sequence

Active site Asp

**Figure S4:** **Multiple sequence alignment of the secreted PLA2 detected in *E. quadricolor* venom with other homologous snake and sea anemone PLA2 proteins.** Alignment was created and visualised using ClustalWS alignment algorithm in Jalview [101]. The active site residues are indicated by blue triangles including the His48/Asp99 dyad [47]. The prepeptide sequence is underlined, the bound Ca^2+^ loop and conserved residues surrounding the dyad active site residues are boxed in black, and the C-terminal extension involved in myotoxic effects is bracketed. The ten conserved cysteine residues are indicated by a blue star and semi conserved cysteine residues are indicated by an orange star . Accession numbers for the homologous anemone and snake PLA2s are indicated in the alignment.


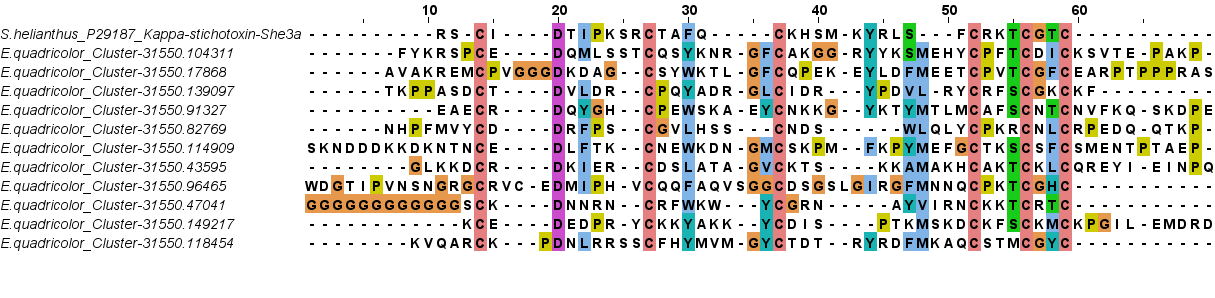


**Figure S5: Alignment of *E. quadricolor* proteins present in venom identified as toxins targeting Type I Kv channels**. Only proteins that contain a single SHK -like domain where aligned with the 35aa SHK peptide potassium channel toxin derived from sun anemone *Stichodactyla helianthus* (P29187) [103]. Alignment was created and visualised using MafftWS alignment algorithm in Jalview and coloured with a Clustal scheme [101]. Blue stars indicate the six conserved cysteine residues that form the three disulfide bonds that are observed in the three-dimensional structure of *S. helianthus* SHK.


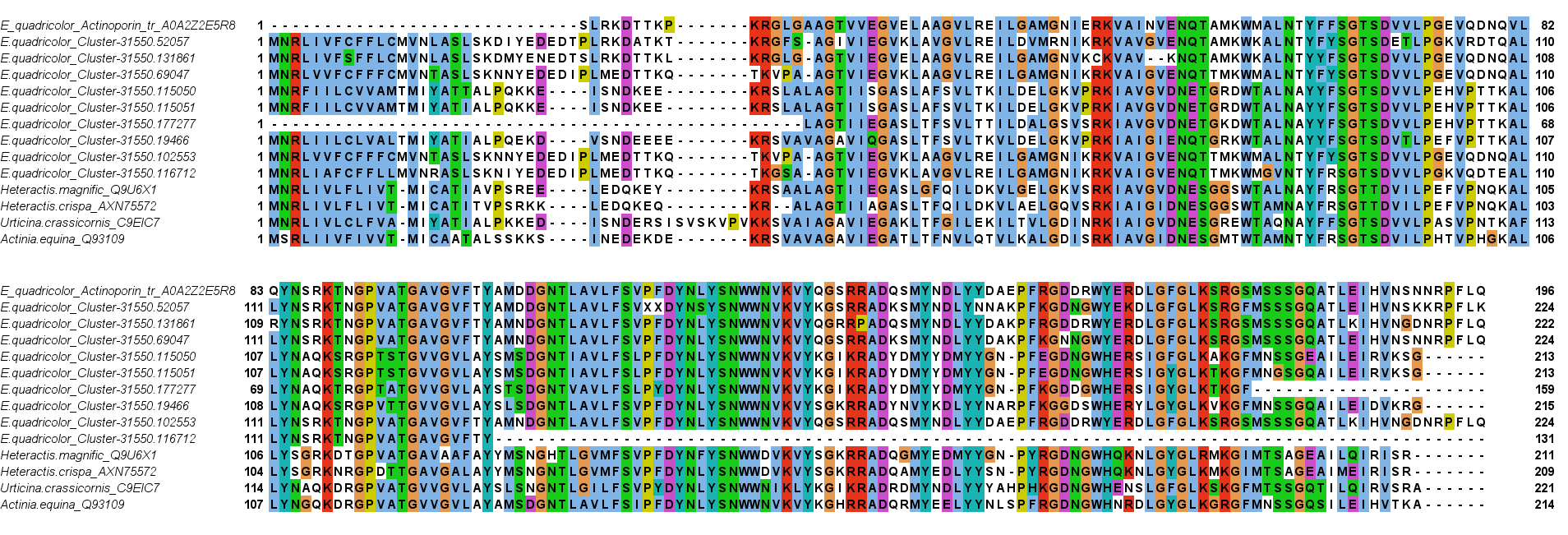


**Figure S6. Multiple sequence alignment of actinoporins and DELTA-actitoxin-Ucs1a proteins detected in *E.quadricolor* venom with homologous actinoporins from host and non-host sea anemones.** Alignment was created and visualised using ClustalWS alignment algorithm in Jalview [101]. Conserved residues that interact with phosphocholine (POC) are highlighted in black boxes and indicated with a blue arrow. The RGD motif is indicated by a star. The red box indicates *E.quadricolor* sequences matching >90% with *E. quadricolor* partial toxin sequence identified by Mebs [48].

### Figure S7 Quality control graphs for RNA sequencing.

**
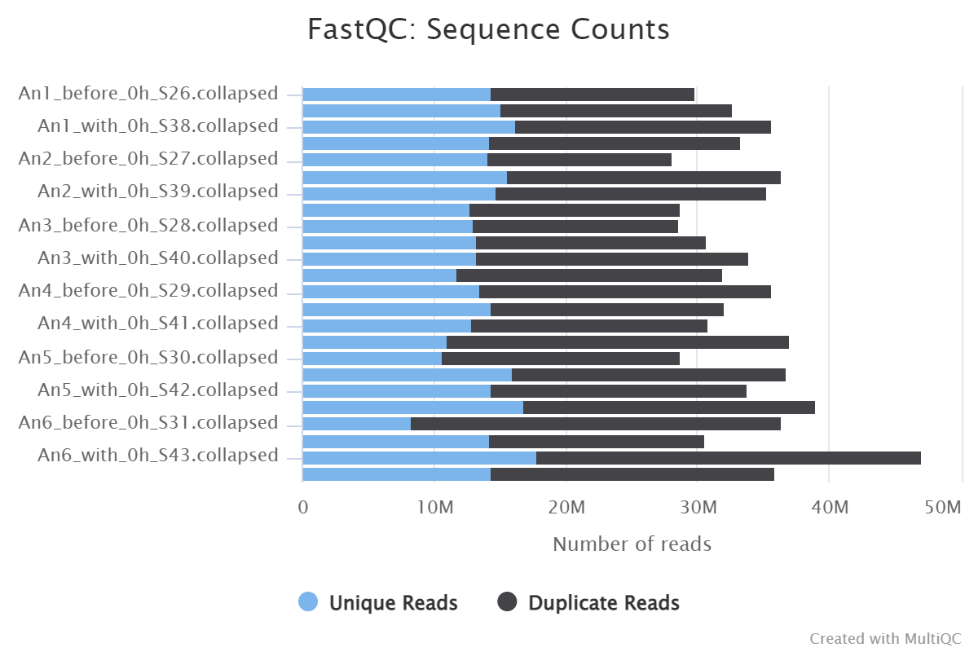
**


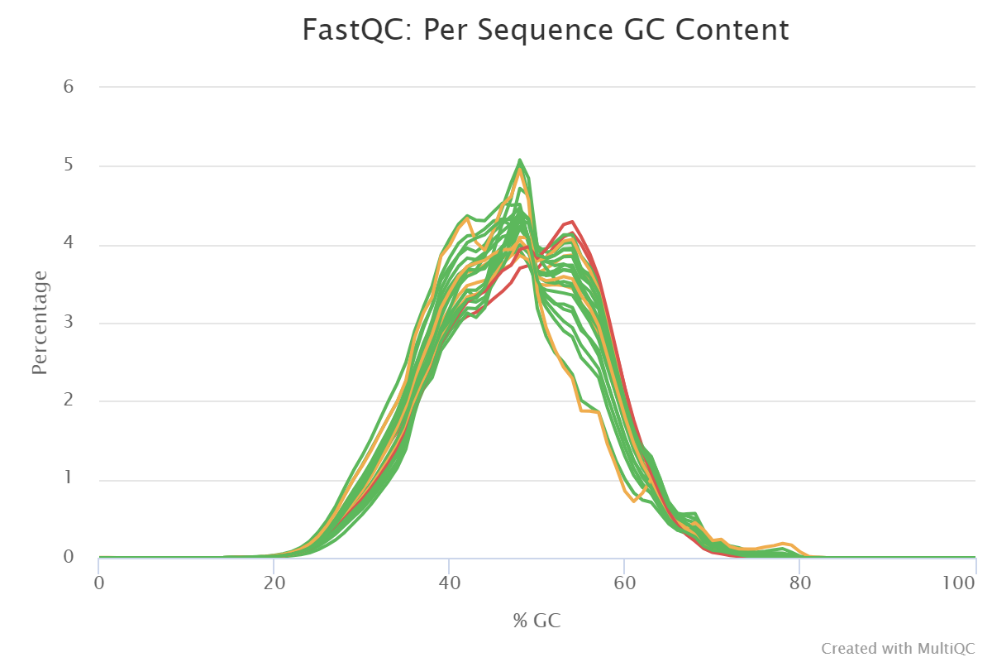


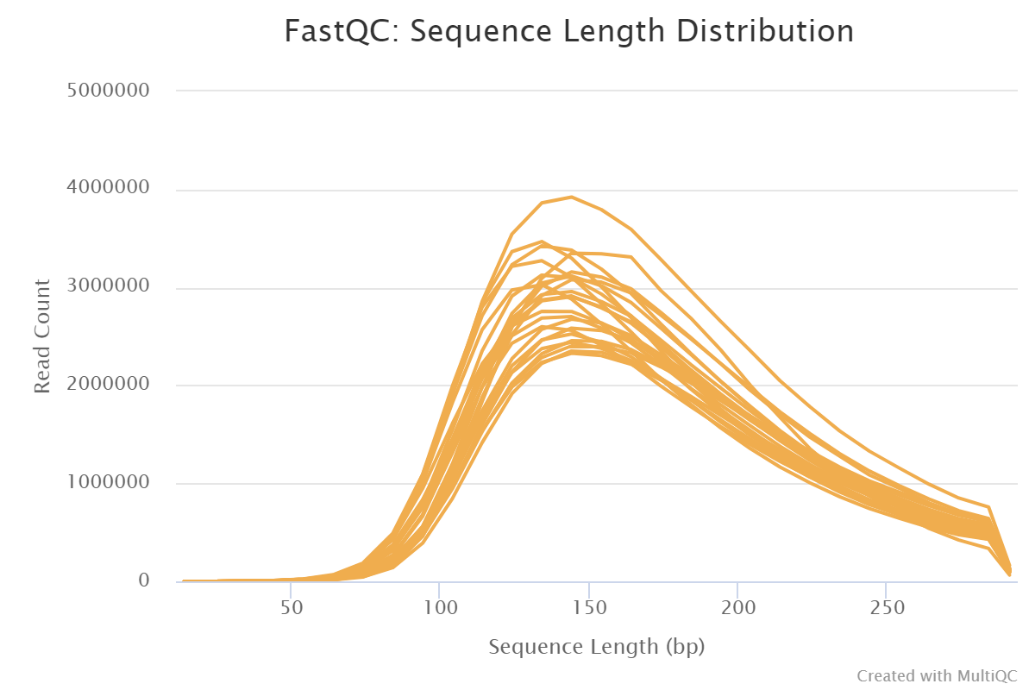

Supplement: Supplementary file 1 [file toxins-16-00085-s001.zip › toxins-2813296-supplementary/supplimentary materials/appendix toxins manuscript ca 050224.docx]
